# A critical review of the role of milk and other dairy products in the development of obesity in children and adolescents

**DOI:** 10.1017/S0954422418000227

**Published:** 2018-11-27

**Authors:** Anestis Dougkas, Suzanne Barr, Sheela Reddy, Carolyn D. Summerbell

**Affiliations:** 1 Institut Paul Bocuse Research Centre, Institut Paul Bocuse, Château du Vivier, BP 25, 69131 Ecully Cedex, France; 2 Department of Medicine, Imperial College London, London, UK; 3 Department of Health, London, UK; 4 Department of Sport and Exercise Sciences, Durham University, Durham, UK

**Keywords:** Dairy products, Milk, Adiposity, Obesity, Children, Adolescents

## Abstract

Existing reviews suggest that milk and other dairy products do not play a role in the development of obesity in childhood, but they do make an important contribution to children’s nutrient intake. It is thus curious that public health advice on the consumption of dairy products for children is often perceived as unclear. The present review aimed to provide an overview of the totality of the evidence on the association between milk and other dairy products, and obesity and indicators of adiposity, in children. Our search identified forty-three cross-sectional studies, thirty-one longitudinal cohort studies and twenty randomised controlled trials. We found that milk and other dairy products are consistently found to be not associated, or inversely associated, with obesity and indicators of adiposity in children. Adjustment for energy intake tended to change inverse associations to neutral. Also, we found little evidence to suggest that the relationship varied by type of milk or dairy product, or age of the children, although there was a dearth of evidence for young children. Only nine of the ninety-four studies found a positive association between milk and other dairy products and body fatness. There may be some plausible mechanisms underlying the effect of milk and other dairy products on adiposity that influence energy and fat balance, possibly through fat absorption, appetite or metabolic activity of gut microbiota. In conclusion, there is little evidence to support a concern to limit the consumption of milk and other dairy products for children on the grounds that they may promote obesity.

## Introduction

### The burden of childhood obesity

Childhood overweight and obesity represent significant short- and long-term challenges for individuals and healthcare, both in Europe and worldwide^(^ ^1^ ^)^. Globally, 41 million children under the age of 5 years were overweight or obese in 2014; 39 % of adults aged 18 years and over were overweight in 2014, and 13 % were obese^(^ ^2^ ^)^. Importantly, the prevalence of obesity is even higher among those children from poor socio-economic backgrounds in most middle- and high-income countries^(^ ^3^ ^)^.

The burgeoning levels of childhood overweight and obesity are a public health concern because childhood obesity is linked to a range of physical and psychological illnesses during childhood^(^ ^4^ ^)^, and tracks through to adulthood^(^ ^5^ ^)^.

Rates of obesity and overweight are increasing globally^(^ ^6^ ^)^. Interventions and public health messages that promote a healthy weight tend to give simple messages to the public^(^ ^7^ ^,^ ^8^ ^)^. These include reducing the consumption of sugar-sweetened beverages and energy-dense snacks, promoting the consumption of fruits and vegetables^(^ ^7^ ^)^, whilst promoting water as a beverage of choice. However, advice on milk and other dairy products is usually unclear and limited, and tends to be perceived as confusing by the public^(^ ^9^ ^)^. Dairy foods play an important role in the diets of children and adolescents but are often under scrutiny because of their energy and fat content^(^ ^10^ ^,^ ^11^ ^)^. For example the UK’s Nutrient Profile Modelling suggests some cheeses and yoghurts are ‘unhealthy’ because there is a focus on fat and saturated fat content with little consideration for micronutrients^(^ ^12^ ^)^.

### Classification of obesity in children

BMI, calculated as weight in kg divided by height in metres squared (kg/m^2^), has been shown to correlate strongly with adiposity (excess body fat) in adults and children, and in turn with health^(^ ^13^ ^)^. We categorise a child’s level of fatness by comparing (plotting) their BMI against age- and sex-specific thresholds (based on centiles) derived from a reference population. In the context of the present review, most studies used BMI as their measure of body fatness, which included BMI, BMI standard deviation score and BMI *z* score. The remaining studies used the outcome measures of percentage body fat, waist circumference and body weight status. We have used ‘obesity and indicators of adiposity’ as a collective term to describe these varying outcome measures.

### Definition of milk and other dairy products

Under European Union regulations, ‘milk’ refers exclusively to the mammary secretion of animals obtained from milking^(^ ^14^ ^)^. The terms milk, whey, cream, butter, buttermilk, cheese and yoghurt are protected and reserved exclusively for dairy products. In the present review, the dairy food group contains milk, cheese and yoghurt, and is described as a group of foods containing Ca, which are to be consumed as a source of Ca^(^ ^15^ ^)^. Butter is not included in the dairy food group as it contains no Ca and is considered to be a fat, and is therefore excluded from the present review. Plant-based milk-like products do not have the same nutrient composition as milk or other dairy products and therefore are not included in this review^(^ ^16^ ^)^. Throughout this publication the terms ‘milk’ and ‘other dairy products’ refer to liquid drinking milk and foods containing Ca which are made from milk such as cheese, yoghurt and fromage frais, ice-cream and dairy desserts (further examples are listed in online Supplementary Table S1)^(^ ^17^ ^)^.

### Contribution of milk and other dairy products to dietary intake

The nutritional value and quality of milk and other dairy products vary across the globe and depend on a number of factors including animal breed, genetic variation within breeds, health and diet of the animal, geographical location of grazing and animal husbandry^(^ ^18^ ^)^. Generally, dairy products are nutrient rich and a source of protein, Ca, riboflavin, iodine, P and vitamin B_12_^(^ ^17^ ^)^; however, their nutrient content varies by type of dairy product (for example, cheese and yoghurt have a different nutrient composition as outlined in online Supplementary Table S2)^(^ ^17^ ^)^.

As defined by European Union regulations, milk is high in protein (defined as having at least 20 % of the energy value of the food provided by protein; 3·6 g per 100 ml), Ca (124 mg per 100 ml), vitamin B_12_ (0·9 µg per 100 ml), riboflavin (0·24 mg per 100 ml) and iodine (31 µg per 100 ml) and is a source of K (160 mg per 100 ml), P (97 mg per 100 ml) and vitamin B_5_ (0·7 mg per 100 ml)^(^ ^17^ ^)^. Whole milk is 3·7 % fat, while skimmed milks are low in fat (0·3 % to 1·8 %)^(^ ^17^ ^)^. Plain milk is low in Na and contains no added sugars^(^ ^17^ ^)^. Cheese is high in protein and full-fat varieties of hard cheeses (but not reduced fat) are a source of vitamin A (387·5 µg per 100 g) and Zn (4·1 mg per 100 g)^(^ ^17^ ^)^ but are considered high in fat. Auestad *et al.* ^(^ ^19^ ^)^ suggest that dairy product intakes make significant contributions to both underconsumed nutrients and overconsumed nutrients^(^ ^19^ ^)^. In developed countries dairy products contribute to over 45 % of children’s Ca intakes, as well as 34 % of their iodine intakes, 20 % of their K intake, 20–40 % of their vitamin B_12_ and 20 % of their vitamin A intake. In terms of overconsumed nutrients, dairy products contribute to just over 10 % of their energy intakes, 10 % of their Na intake and 20–24 % of their saturated fat intake^(^ ^19^ ^)^.

### Consumption trends in milk and other dairy products

Milk consumption varies around the world, from 10 to 212·2 kg per capita per year^(^ ^20^ ^)^. Industrialised countries consume the most milk at 212·2 kg/year and East Asian countries consume the least at 10 kg/year^(^ ^20^ ^)^. Data from the Global Nutrition and Policy Consortium show that younger children consume more milk than older children (338·1 and 147·2 g/d, respectively)^(^ ^21^ ^)^.

## Existing review-level evidence from observational and intervention studies of the impact of milk and other dairy products on childhood and adolescent obesity and indicators of adiposity

The association between dairy products and body composition has been widely reviewed with data from both observational and intervention studies, of different study designs, in children and in adolescents. Examples of reviews and meta-analyses include Louie *et al.* ^(^ ^22^ ^)^, Dror & Allen^(^ ^23^ ^)^, Dror^(^ ^24^ ^)^, Lu *et al.* ^(^ ^25^ ^)^, Wang *et al.* ^(^ ^26^ ^)^ and Kouvelioti *et al.* ^(^ ^27^ ^)^. Examining the evidence by study design, a recent meta-analysis of mainly (but not exclusively) seven cross-sectional studies by Wang *et al.* ^(^ ^26^ ^)^ found that intake of total dairy products was associated with a decreased risk of obesity in children (pooled OR 0·54; 95 % CI 0·38, 0·77). However, this pooled meta-analysis included mainly cross-sectional studies, which are susceptible to reverse causality and conducted in Asian populations with high heterogeneity between the studies. A recent meta-analysis of ten prospective cohort studies found that children in the highest dairy product intake group at baseline were 38 % less likely to be overweight or obese over time relative to those who were in the lowest group^(^ ^25^ ^)^. An increase of one serving/d of dairy products was linked with a 0·65 % lower body fat and a 13 % lower risk of overweight/obesity. However, another recent systematic review of randomised intervention trials^(^ ^27^ ^)^ showed that eleven out of the fourteen studies demonstrated a neutral effect of milk consumption on body composition in children and adolescents, highlighting the difference in the results based on the type of study.

The most comprehensive review to date is a meta-analysis of combined results from twenty-two studies selected by the authors, which included cross-sectional, prospective cohort and intervention studies^(^ ^24^ ^)^. This review found no association between dairy product consumption and adiposity in preschoolers and school-aged children, although there was an inverse association between dairy product intake and adiposity in adolescents (average effect size −0·26 (95 % CI −0·38, −0·14); *P*<0·0001). However, this meta-analysis had a few deficiencies and there was strong evidence of high study heterogeneity as seen in the *I* ^2^ statistics (*I* ^2^=0·72). The difficulty in comparing or combining results from different studies is due to the high heterogeneity in terms of exposures (type of dairy products or serving portion) and outcomes (BMI, weight change or adiposity).

The existing evidence, as summarised in the above reviews and meta-analyses, shows consistently that milk or dairy product consumption in children either has no relationship with weight status, or has an inverse relationship with excess weight gain (also reported in various ways). However, there were only a limited number of studies included in the meta-analyses due to the diversity and high heterogeneity between studies, which are often susceptible to risk of bias and inconsistency and also publication bias^(^ ^28^ ^)^. Therefore, we argue that a critical and up-to-date evaluation of the available scientific evidence on this topic is lacking. First, the existing reviews are not comprehensive in their inclusion of available evidence from different study designs. Examination of individual studies contributing to narrative summaries will enable us to have more complete reflection and wide overview of studies dealing with dairy products and obesity and indicators of adiposity. Second, it remains unclear whether the associations reported relate equally to all dairy products, milk and other dairy products, as none of the systematic reviews or meta-analyses reported differences between different types of milk and/or other dairy products, and obesity and indicators of adiposity, in children and adolescents. Third, it is unclear whether the associations reported changed during childhood.

### Scope of the review

The present review aims to provide an overview of the totality of the available evidence taken from cross-sectional studies, prospective longitudinal studies and intervention studies that have reported on the associations between intakes of milk and other dairy products, and obesity and indicators of adiposity, in children. This critical review also aims to explore whether any associations identified related equally to all milk and other dairy products. It also provides context for these findings, specifically in terms of the mechanisms by which milk and other dairy products might make an impact on obesity and indicators of adiposity in children and adolescents.

## Research methodology

**Fig. 1:**
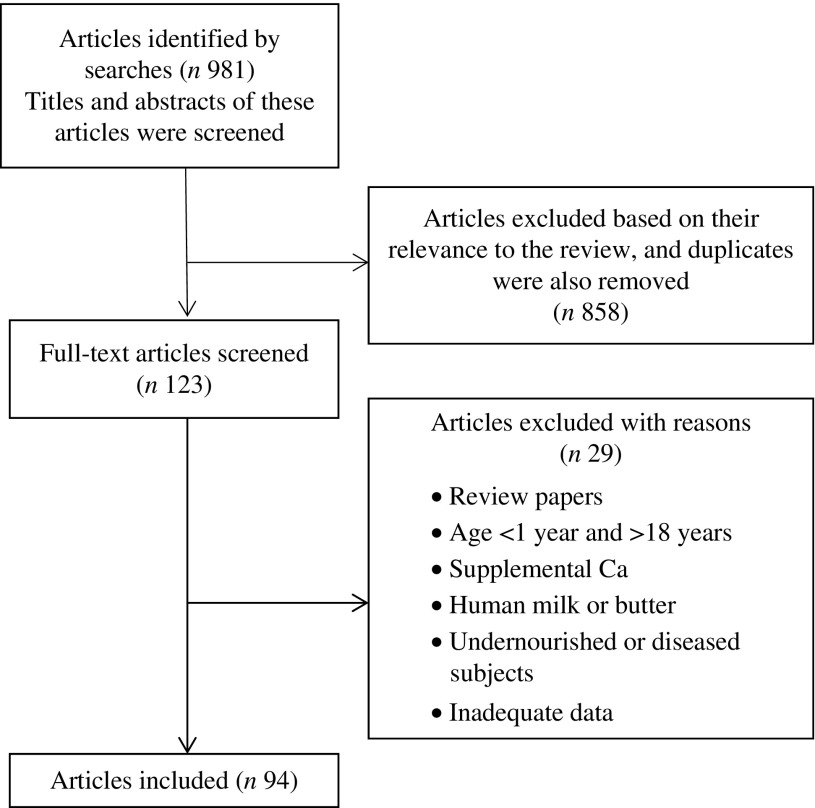
Flow diagram for the selection of eligible studies.

The literature for English-language articles published from January 1990 up to June 2017 was reviewed by searching the following three databases: ISI Web of Knowledge/Web of Science (Thomson Reuters, UK), PubMed (US National Library of Medicine National Institutes of Health) and Google Scholar (Google, UK). Search terms included ‘dairy’, ‘milk’, ‘cheese’, ‘yoghurt’, ‘calcium’, ‘obesity’, ‘adiposity’, ‘overweight’, ‘body fatness’, ‘body weight’, ‘children’, ‘adolescent’, ‘girls’, ‘boys’ and combinations of these. In addition, reference lists of identified published articles as well as of previous narrative, systematic reviews and meta-analyses were searched for citations of relevant publications. A total of 981 hits were retrieved. All observational and intervention studies conducted in adults, in non-healthy children and those which assessed body weight in infants (<1 year) were not included in this review. Studies that examined exclusively Ca supplements in relation to childhood or adolescent obesity were also excluded. However, studies that compared directly the effect of dairy products or dietary Ca with Ca supplements on adiposity were included, as they provide a better understanding of the putative functional properties of bioactive constituents in milk and other dairy products. Studies that examined exclusively butter were not included, as according to the United States Department of Agriculture, any milk products with little to no Ca are not included in the dairy food group^(^ ^15^ ^)^. Studies that had bone density as the primary outcome but included body weight or measures of body composition were included in the present review. Using these selection criteria, a total number of forty-three cross-sectional studies, thirty-one longitudinal cohort studies and twenty intervention (randomised controlled trial) studies were identified that examined the association/impact of dairy milk and other dairy products on childhood and adolescent obesity and indicators of adiposity. Fig. 1 summarises the literature search strategy.

Using the same three databases and predefined publication dates, the search terms ‘dairy’, ‘milk’, ‘calcium’, ‘mechanisms’, appetite’, ‘fat oxidation’, ‘fat metabolism’, ‘gut microbiota’, ‘energy intake’, ‘satiety’, ‘children’, ‘adolescent’, ‘girls’, ‘boys’ and combinations of these were used for the studies mentioned in the mechanistic section of the present review. Mechanistic studies in cells or animals were limited to searches of the reference lists of identified published studies and relevant reviews for citation of relevant publications.

We have summarised the evidence base, which we identified by study design; cross-sectional studies, longitudinal studies, and intervention studies and provided a narrative summary for the studies we identified by study design. As this is not a systematic review, we did not conduct a meta-analysis by study design but summarised the findings for each study in Tables 1 and 2 and online Supplementary Table S3. Symbols ↑ and ↓ indicate a statistically significant positive and negative effect/relationship, respectively, between dairy milk and other dairy products and obesity and indicators of adiposity, with ↔ indicating no statistically significant effect/relationship or a neutral effect. Such a simple tabulated summary allowed us to draw overall conclusions for this narrative review, although we did not undertake a detailed assessment of quality of individual studies. We have learnt from other reviews we have published that this type of simple tabulated summary can be helpful for the broad range of readers who may be interested in the work. However, it is important to note that it is not appropriate to ‘add up the arrows’ within each study design to assess the balance of evidence since this would ignore sample size, statistical power of each study, and how heavily each study should be weighted in considering the overall evidence base. For example, ten ↑ studies plus one ↓ study plus five ↔ studies cannot be interpreted as a summary finding of a statistically significant positive effect/relationship between dairy milk and other milk products and obesity. We appreciate that if we had conducted a systematic review and assessed each included study for quality (based on design and implementation), and combined the results in a meta-analysis, then each individual study’s findings would contribute to the overall (summary) effect size, and this would be an appropriate way to assess the balance of evidence.

**Table 1:** Prospective studies (*n* 31) that measured milk and other dairy product consumption at baseline and change in body composition over time in children

Reference	Details	Exposure	Results and conclusion	Adjustment	Effect
Carruth & Skinner (2001)^(^ ^45^ ^)^	*n* 53 (29 boys) Age: 2–2·3 years, about 6 years follow-up USA	Dietary Ca and total dairy products	Average dairy product consumption over the years was associated with lower %BF (*β* –3·54 (se 1·04); *P*=0·001) and BF g (*β* –907·06 (se 284·06); *P*=0·003)	Sex, BMI, Ca, protein, carbohydrates and fat	↓
Skinner *et al.* (2003)^(^ ^46^ ^)^	*n* 52 (25 boys) Age: 2–2·3 years, 8-year follow-up USA	Dietary Ca	Average dietary Ca intake over the years was associated with lower %BF in various models (*P*=0·02 to 0·04)	Sex, total fat, sedentary activity	↓
Phillips *et al.* (2003)^(^ ^33^ ^)^	*n* 196 (girls) Age: 8–12 years, 4 years post-menarche USA	Dairy Ca and total dairy products	Dairy product consumption and Ca consumption were not associated with BMI *z* score or %BF during adolescence (*P*>0·05)	Physical activity, energy intake, parental overweight, protein, soda, fruit and vegetables	↔
Fisher *et al.* (2004)^(^ ^48^ ^)^	*n* 192 (girls) Age: 5–9 years, 4-year follow-up USA	Dietary and supplementary Ca	There was no difference in BMI *z* score from age 5 to 9 years between the girls who met and those who fell below the recommended adequate intake for Ca (*P*=0·83) despite the higher energy intake in those who met the recommendation	Pubertal status	↔
Rockell *et al.* (2004)^(^ ^50^ ^)^	*n* 46 (18 boys) Age: 6–10 years, 2-year follow-up New Zealand	Milk	Non-milk consumers had higher BMI *z* scores at baseline and 33 % of the children were overweight or obese at the 2-year follow-up compared with the reference population (*P*<0·01)	Unadjusted	↓
Newby *et al.* (2004)^(^ ^165^ ^)^	*n* 1345 (675 boys) Age: 2–5 years, 8-month follow-up USA	Milk	There was no association between milk consumption and annual change in weight and BMI *z* scores (*β* 0·00 (se 0·01); *P=*0·84)	Age, sex, birth weight, energy intake, sociodemographic variables, height change	↔
Dixon *et al.* (2005)^(^ ^47^ ^)^	*n* 342 (171 boys) Age: 4–10 years, 1-year follow-up USA	Dietary Ca and total dairy products	There were no associations between dietary Ca intake over a year and measures of adiposity in all age groups in hypercholesterolaemic children (*P*>0·05). However, Ca intake was inversely associated with BMI, sum of skinfolds and trunk skinfolds in the 7- to 10-year-old non-hypercholesterolaemic children	Age, sex, time period, energy intake and fat	↔ (HC) ↓ (Non-HC)
Berkey *et al.* (2005)^(^ ^41^ ^)^	*n* 12 829 (5550 boys) Age: 9–14 years, 4-year follow-up USA	Milk (full fat, 2 % and 1 % fat)	High milk consumption (≥3 servings/d) was associated with higher BMI compared with low milk consumption (≤0·5 servings/d) in both boys (*β* 0·08 (se 0·04); *P=*0·04) and girls (*β* 0·09 (se 0·03); *P=*0·007)	Age, dietary factors, ethnicity, height growth, prior BMI *z* score, Tanner stage, menarche, physical activity and inactivity	↑
Faith *et al.* (2006)^(^ ^166^ ^)^	*n* 971 (517 boys) Age: 1–5 years, 4-year follow-up Australia	Milk (2 % or whole fat)	There was no association between increased milk intake and excess adiposity gain (BMI *z* score of β –0·002 (se 0·002); *P*=0·39)	Baseline child’s weight-for-height *z* score, sex, race/ethnicity, children’s food and beverage intake, parental feeding styles and attitudes variables	↔
Moore *et al.* (2006)^(^ ^55^ ^)^	*n* 92 (56 boys) Age: 3–6 years, 8-year follow-up USA	Total dairy products	Low dairy product consumption (<1·75 servings/d) was associated with greater subcutaneous fat (25 mm; *P*=0·005) and higher BMI (2 units; *P*=0·046) by the time of early adolescence compared with high dairy product consumption	Age, physical activity, maternal education, baseline anthropometry, saturated fat, energy intake	↓
Striegel-Moore *et al.* (2006)^(^ ^51^ ^)^	*n* 2371 (girls) Age: 9–10 years, 10-year follow-up USA	Milk	BMI decreased by *β* –0·002 (se 0·006) units for every 100 g of milk (plain or flavoured) although not statistically significant (*P*>0·05)	Race, site, visit, other beverages and energy intake	↔
Tam *et al.* (2006)^(^ ^167^ ^)^	*n* 281 (141 boys) Age: mean 7·7 (sd 0·6) years, 4·0–6·6 years follow-up Australia	Milk	There was no association between milk consumption at baseline and BMI status at follow-up (*P*=0·995)	Unadjusted	↔
Johnson *et al.* (2007)^(^ ^168^ ^)^	*n* 362, age 5 years *n* 471, age 7 years, 2–4 years follow-up UK	Milk	There was an inverse association between milk consumption at 5 or 7 years of age (–0·51 (95 % CI –0·86, –0·16); *P*<0·01) and fatness at 9 years (–0·35 (95 % CI –0·57, –0·14); *P*<0·01)	Age, sex, BMI at baseline, height at 9 years, television viewing, maternal education, paternal class and BMI, energy intake misreporting, energy density, dietary factors	↓
Günther *et al.* (2007)^(^ ^139^ ^)^	*n* 203 (102 boys) Age: 6 months, 7-year follow-up Germany	Dairy protein	Higher dairy protein consumption (% of energy) at age 12 months was positively associated with BMI-SDS at 7 years of age (T1: 0·03 (95 % CI –0·21, 0·27) *v.* T3: 0·35 (95 % CI 0·13, 0·57); *P*=0·02)	Sex, maternal overweight, maternal education, protein, fat, fibre, energy intake, siblings in the dataset, firstborn status, smoking in the house, baseline BMI-SDS and %BF	↑
Barr (2007)^(^ ^49^ ^)^	*n* 45 (girls) Age: mean 10·5 (sd 0·6) years, 2-year follow-up Canada	Dietary and supplementary Ca	There was no association between Ca consumption and 2-year changes in %BF and % trunk fat in girls (*P*>0·05)	Unadjusted	↔
Kral *et al.* (2008)^(^ ^169^ ^)^	*n* 49 Age: 3–6 years, 3-year follow-up USA	Energy consumed from milk at ages 3–5 years	Greater increases in energy consumed from milk were inversely related to changes in children’s waist circumference (β –0·01 (se 0·004); *P*=0·04)	Change in waist circumference from ages 3 to 5 years and total energy intake at age 3 years	↓
Fiorito *et al.* (2009)^(^ ^170^ ^)^	*n* 170 (girls) Age: 5 years, 10-year follow-up USA	Milk	Milk consumption at 5 years of age was not associated with greater adiposity or weight status over a 10-year period (*P*>0·05)	Unadjusted	↔
Huus *et al.* (2009)^(^ ^171^ ^)^	*n* 14 224 (6866 boys) Age: 2·5 years, 2·5-year follow-up Sweden	Cream/crème fraîche, cheese, ice-cream	Cheese consumption at 2·5 years of age was positively while cream/crème fraîche was inversely associated with overweight or obesity at 5 years of age	Mother’s education and BMI, father’s education and BMI, heredity of diabetes, vegetables, potatoes, fried potatoes, eggs, sausage, chocolate, candies, porridge	↑ (Cheese) ↓ (Cream)
Vanselow *et al.* (2009)^(^ ^36^ ^)^	*n* 2294 (1032 boys) Age: mean 14·9 (sd 0·1) years, 5-year follow-up USA	Milk	There was an inverse association between milk consumption and mean weight gain over a 5-year period without displaying a dose–response relationship (0 servings/week=2·34 (se 0·24) kg *v.* 0·5–6 servings/week=1·68 (se 0·11) kg *v.* >7 servings/week=1·93 (se 0·11) kg; *P*=0·03)	Age, sex, race/ethnicity, socio-economic status, baseline BMI, physical activity, television viewing, beverage, coffee and tea intake	↓
Huh *et al.* (2010)^(^ ^39^ ^)^	*n* 852 (466 boys) Age: 2 years, 1-year follow-up USA	Dairy products and milk (full-fat and reduced-fat)	There was no association between dairy product or milk (either full- or reduced-fat) consumption at 2 years of age and BMI *z* score or with the risk of incident overweight at age 3 years (*P*>0·05)	Age, sex, race/ethnicity, baseline BMI *z* score, energy intake, non-dairy beverage intake, television viewing, maternal BMI and education, paternal BMI	↔
Noel *et al.* (2011)^(^ ^38^ ^)^	*n* 2245 (1030 boys) Age: 10 years, 1–3 years follow-up UK	Milk (full-fat and reduced-fat)	There was no association between milk consumption at age 10 years and BF at 13 years of age (*β* –0·15 (95 % CI –0·52, 0·23); *P*=0·45). Changes in milk consumption from 10 to 13 years of age were not associated with changes in %BF from 11 to 13 years of age	Age, sex, height, physical activity, pubertal status, maternal BMI and education, fat, sugar-sweetened beverages, cereals, energy intake and baseline BMI	↔
Garden *et al.* (2011)^(^ ^32^ ^)^	*n* 362 (183 boys) Age: 18 months, 8-year follow-up Australia	Total dairy products	Higher consumption of dairy products (% of total energy) was inversely associated with BMI at 8 years (*β* –0·21 (95 % CI –0·41, 0·01); *P*=0·04)	Sex, asthma study intervention group, birth weight, breast-feeding for 6 months, parental obesity status, ethnicity, smoking in pregnancy, paternal education	↓
Lin *et al.* (2012)^(^ ^172^ ^)^	*n* 5968 (2900 boys) Age: 11 years, 2-year follow-up China	Milk	There was no association between milk consumption at 11 years and BMI *z* score at approximately 13 years of age (*β* –0·01 (95 % CI –0·07, 0·05); *P*=0·65)	Sex, baseline BMI *z* score, birth order, maternal age and mother’s birthplace, highest parental education, physical activity, vegetable, fruit and soft drink intakes	↔
Rangan *et al.* (2012)^(^ ^31^ ^)^	*n* 335 (169 boys) Age: 16–24 months, 7·7–9·2 years follow-up Australia	Total dairy products	There was no association between dairy product consumption at 18 months and BMI at age 8 years (*P*=0·09)	Unadjusted	↔
Noel *et al.* (2013)^(^ ^35^ ^)^	*n* 2270 (1028 boys) Age: 10 years, 1–3 years follow-up UK	Flavoured milk	Flavoured milk consumption at age 10 years was associated with smaller reduction in %BF in overweight/obese children from ages 11 to 13 years compared with the non-flavoured milk consumption (–0·79 (95 % CI –2·46, –0·88) *v.* –2·19 (95 % CI –3·60, –0·78); *P*=0·02, respectively)	Age, sex, height, height^2^, baseline BMI, physical activity, pubertal status, maternal BMI and education, fat, cereal, fruit, vegetable, sugar-sweetened, milk and energy intakes	↔
Scharf *et al.* (2013)^(^ ^40^ ^)^	*n* 8300 (4200 boys) Age: 4 years, 2-year follow-up USA	Milk (full-fat and reduced-fat)	There was no association between either full- or reduced-fat milk at age 4 years in the change in BMI *z* score over the 2-year follow-up (*P*=0·6)	Sex, race/ethnicity, socio-economic status	↔
Hasnain *et al.* (2014)^(^ ^53^ ^)^	*n* 103 Age: 3–5 years, 12-year follow-up USA	Milk	Higher milk consumption at ages 3–9 years was negatively associated with %BF at ages 15–17 years (T1, 30 % *v.* T3, 22·6 %; *P*=0·009)	Age, baseline anthropometry, fat, television viewing, beverage intake, maternal BMI and education	↓
DeBoer *et al.* (2014)^(^ ^54^ ^)^	*n* 8950 (4550 boys) Age: 4 years, 1-year follow-up USA	Milk	There was no association between milk consumption at 4 years and BMI *z* score (*P*=0·79) or weight-for-height *z* score (*P*=0·24) at age 5 years	Sex, race/ethnicity, socio-economic status and milk type	↔
Bigornia *et al.* (2014)^(^ ^42^ ^)^	*n* 2455 (1154 boys) Age: 10 years, 3-year follow-up UK	Dairy products (full-fat and reduced-fat)	High full-fat dairy product consumption at age 10 years was associated with lower risk of total BF mass at 13 years (OR 0·64 (95 % CI 0·40, 1·00); *P*=0·04)	Age, sex, height, total dairy products at 13 years, adiposity at 10 years, maternal education and overweight status, physical activity, pubertal stage and dieting	↔ (Dairy products) ↓ (Full-fat dairy products)
Braun *et al.* (2016)^(^ ^140^ ^)^	*n* 3564 (1748 boys) Age: 12 months, 8-year follow-up Netherlands	Dairy protein	A 10 g higher total dairy protein intake/d at 1 year was associated with a 0·07-sd increase in weight (95 % CI 0·03, 0·012; *P*<0·05) and a 0·07-sd increase in BMI (95 % CI 0·02, 0·11) at 9 years. Similar effect size for non-dairy food sources	Birth weight *z* score, breast-feeding, playing sports, household income, and maternal BMI at enrolment, education, folic acid use during pregnancy, smoking during pregnancy and non-dairy animal protein	↑
Dubois *et al.* (2016)^(^ ^43^ ^)^	*n* 152 twin pairs Age: 9 years, 5-year follow-up Canada	Milk (full-fat and reduced-fat)	Milk consumption at 9 years was positively associated with BMI change from 9 to 14 years. Reduced-fat milk was positively associated with BMI change in girls	Unadjusted	↑ (Milk) ↑ (Reduced-fat milk for girls)

%BF, percentage body fat; BF, body fat; ↓, negative association between exposure (dairy products) and a measure of body fatness; ↔, null association between exposure (dairy products) and a measure of body fatness; HC, hypercholesterolaemic; non-HC, normocholesterolaemic; ↑, positive association between exposure (dairy products) and a measure of body fatness; SDS, standard deviation score; T1, tertile 1; T3, tertile 3.

**Table 2:** Randomised intervention studies (*n* 20) of milk and other dairy product consumption on weight gain or body composition in children

Reference	Details	Intervention	Results and conclusion	Adjustment	Effect
Chan *et al.* (1995)^(^ ^67^ ^)^	*n* 48 (girls) Age: 9–13 years, 12 months USA	Diets: (a) Dairy products (1200 mg Ca/d) (b) Control (usual diet)	Increased dairy product consumption over a year was not associated with weight gain or increased %BF	Unadjusted	↔
Cadogan *et al.* (1997)^(^ ^68^ ^)^	*n* 82 (girls) Age: mean 12·2 (sd 0·3) years, 18 months UK	Diets: (a) 568 ml milk/d (b) Control (usual diet)	Increased milk consumption over 18 months was not associated with increased height, weight, LBM and BF	Unadjusted	↔
Merrilees *et al.* (2000)^(^ ^69^ ^)^	*n* 91 (girls) Age: 15–16 years, 2-year intervention and 1-year follow-up New Zealand	Diets: (a) Dairy products (1000 mg Ca/d) (b) Control (usual diet)	There were no differences in height, weight, BF and LBM between the two groups after 2 years and 1-year follow-up (*P*>0·05)	Unadjusted	↔
Volek *et al.* (2003)^(^ ^70^ ^)^	*n* 28 (boys) Age: 13–17 years, 3 months USA	Diets: (a) RT and 1 % milk (3 servings/d) (b) RT and fruit juice (3 servings/d)	There were no differences in body mass, LBM, fat mass and %BF over the 3 months between the milk and fruit juice groups (*P*>0·05)	Unadjusted	↔
Lappe *et al.* (2004)^(^ ^76^ ^)^	*n* 59 (girls) Age: 9 years, 2 years USA	Diets: (a) Ca-rich diet (1500 mg/d) (b) Habitual diet	There was no difference in change of height, weight, BMI, fat mass or LBM between the Ca-rich diet (mainly from dairy products) and the habitual diet over 2 years	Unadjusted	↔
Du *et al.* (2004)^(^ ^75^ ^)^	*n* 757 (girls) Age: 10 years, 2 years China	Diets: (a) 330 ml milk/school day (b) 330 ml milk and vitamin D_3_/school day (c) Control (usual diet)	High milk consumption over the 2 years increased body weight (2·9 % for milk and 3·7 % for milk and vitamin D_3_ group; *P*<0·0005) compared with the control group (0·8 %; *P*=0·3)	Baseline	↑
Lau *et al.* (2004)^(^ ^72^ ^)^	*n* 344 (189 boys) Age: 9–10 years, 18 months China	Diets: (a) Milk powder (40 g/d) (b) Milk powder (80 g/d) (c) Control (usual diet)	There was no difference in change of height, weight, LBM and fat mass between either the 40 or 80 g milk supplementation and the control (*P*>0·05)	Sex, Tanner stage, baseline values, baseline Ca, protein, physical activity and time to follow-up	↔
Gibbons *et al.* (2004)^(^ ^173^ ^)^	*n* 123 (75 boys) Age: 8–10 years, 12-month intervention, 12-month follow-up New Zealand	Diets: (a) Dairy products (80 mg milk powder, 1200 mg Ca/d) (b) Placebo (80 mg powder, 400 mg Ca/d)	There was no difference in change of height, weight, LBM and fat mass between the intervention and the placebo group (*P*>0·05)	Unadjusted	↔
Cheng *et al.* (2005)^(^ ^71^ ^)^	*n* 195 (girls) Age: 10–12 years, 2-year follow-up Finland	Diets: (a) Ca (1000 mg) and vitamin D_3_ (b) Ca (1000 mg) (c) Cheese (1000 mg Ca) (d) Placebo (e) Habitual diet (>900 mg Ca)	There were no differences in changes in body weight, fat mass, LBM and height between the groups after 2 years (*P*>0·05)	Unadjusted	↔ (Ca) ↔ (Cheese)
DeJongh *et al.* (2006)^(^ ^174^ ^)^	*n* 178 (93 boys) Age: 3–5 years, 1 year USA	Diets: (a) Diet and 1000 mg supplementary Ca (b) Diet and placebo (0 mg Ca)	There was no difference in changes in fat mass or %BF and dietary Ca (*r* –0·01, *P*=0·9 and *r* –0·05, *P*=0·5) or total (dietary and supplementary) Ca intake (*r* –0·02, *P*=0·8 and *r* –0·06, *P*=0·4)	Unadjusted	↔
Albala *et al.* (2008)^(^ ^80^ ^)^	*n* 98 (52 boys) Age: 8–10 years, 4 months China	Diets: (a) Milk (3 servings/d) and no sugar-sweetened beverages (b) Control (usual diet)	There was no difference in change of total fat mass between the intervention and control groups, yet LBM remained higher (*P*=0·04) following the intervention (0·92 (se 0·10) kg) than in the control group (0·62 (se 0·11) kg)	Age, sex	↔ (BF)
Ghayour-Mobarhan *et al.* (2009)^(^ ^86^ ^)^	*n* 96 Age: 12–18 years, 3 months Iran	Diets: (a) 500 kcal (2090 kJ)/d deficit and dairy products (2 servings/d) (b) 500 kcal (2090 kJ)/d deficit and dairy products (3 servings/d) (c) 500 kcal (2090 kJ)/d deficit and dairy products (4 servings/d)	Increased dairy product consumption over 3 months was not associated with augmented BMI, BMI *z* score, weight, %BF and BF mass in overweight and obese children under an energy-restricted diet	Age, sex, ethnicity	↔
St-Onge *et al.* (2009)^(^ ^82^ ^)^	*n* 45 (9 boys) Age: 8–10 years, 4 months USA	Diets: (a) High milk (4 × 236 ml/d) (b) Low milk (1 × 236 ml/d)	There were no differences in change of body weight or body composition between the high- and low-milk consumption groups in overweight children over 16 weeks	Unadjusted	↔
Kelishadi *et al.* (2009)^(^ ^83^ ^)^	*n* 95 Age: mean 5·6 (sd 0·5) years, 6-month intervention, 3-year follow-up (assessed twice yearly) Iran	Diets: (a) Isoenergetic dairy-food-rich diet (>800 mg Ca/d) (b) Energy-restricted diet (c) Control (no dietary recommendations)	There were no differences in BMI-SDS, %BF and WC between the three groups after the 6-month intervention, yet the high dairy product group had lower increases in BMI-SDS and WC than the energy-restricted and control groups over the follow-up (*P*=0·04)	Unadjusted	↔ (Intervention) ↓ (Follow-up)
Weaver *et al.* (2011)^(^ ^90^ ^)^	*n* 42 (17 boys), overweight Age: 12–15 years, 3 weeks USA	Diets: Cross-over design (a) Intervention (1300 mg Ca/d) (1) CaCO_3_ (50 % children) (2) Dairy Ca (50 % children) (b) Control (650 mg diet Ca/d)	There were no differences in changes in body weight between the control and intervention sessions (from either CaCO_3_ or dairy Ca) in adolescent overweight boys and girls (*P*=0·6)	Unadjusted	↔
Arnberg *et al.* (2012)^(^ ^78^ ^)^	*n* 203 (120 boys) Age: 12–15 years, 3 months Denmark	Diets: (a) Pre-test control (b) Water (c) Casein (35 g protein/l) (d) Skimmed milk (1 litre/d) (e) Whey (35 g protein/l)	High consumption of skimmed milk, whey and casein for 3 months increased BMI-for-age *z* scores and WC compared with pre-test control and water groups in overweight adolescents (*P*<0·05)	Age, sex, pubertal stage	↑
Larnkjaer *et al.* (2014)^(^ ^79^ ^)^	*n* 193 (120 boys) Age: 12–15 years, 3 months Denmark	Diets (a) Pre-test control (b) Water (c) Casein (35 g protein/l) (d) Skimmed milk (1 litre/d) (e) Whey (35 g protein/l)	High consumption of skimmed milk, whey and casein for 3 months increased FMI compared with water (*P*<0·05), whilst all four groups increased LMI more than the pre-test control group in overweight adolescents (*P*< 0·05)	Age, sex, pubertal stage	↑
Weber *et al.* (2017)^(^ ^73^ ^)^	*n* 139 (86 girls) Age: 7–10 years, 36 months USA	Diets: (a) Intervention (behavioural modification and nutritional education (1500 mg diet Ca/d)) (b) Control (usual-care group)	High consumption of dietary Ca was associated with lower increases in BMI (*β* –0·0012 (95 % CI –0·002, –0·001); *P*=0·007) and FMI (*β* –0·001 (95 % CI –0·002, –0·001); *P*=0·02) at 24 months in both intervention and control groups	Age, sex, African American ancestry group	↓
Lappe *et al.* (2017)^(^ ^81^ ^)^	*n* 274 (girls) Age: 13–14 years, 12 months USA	Diets: (a) Dairy products (≥1200 mg Ca/d) (b) Control (usual diet, ≤600 mg Ca/d)	There were no differences in change of BF (*P*=0·45) or weight gain (*P*=0·58) between the groups in overweight adolescent girls over 12 months	Unadjusted	↔
Vogel *et al.* (2017)^(^ ^74^ ^)^	*n* 240 (86 boys) Age: 8–16 years, 18 months USA	Diets: (a) Dairy products (additional 3 servings/d) (b) Control (usual low-dairy food diet)	There were no differences in change of BF gain by increasing dairy product consumption to 3 servings/d (1500 mg Ca/d) compared with 2 servings/d (1000 mg Ca/d) independent of weight status, sex or race over 18 months	Sex, race, study site, Tanner stage	↔

%BF, percentage body fat; ↔, null association between exposure (dairy products) and a measure of body fatness; LBM, lean body mass; BF, body fat; RT, resistance training; ↑, positive association between exposure (dairy products) and a measure of body fatness; SDS, standard deviation score; WC, waist circumference; ↓, negative association between exposure (dairy products) and a measure of body fatness; FMI, fat mass index; LMI, lean mass index.

## Evidence from cross-sectional studies (*n* 43) of the associations between dairy product intake and body weight in children and adolescents

As a starting point to our research, and also for completeness, we reviewed the evidence reported from cross-sectional studies (online Supplementary Table S3). Forty-three cross-sectional studies were identified for this review. Studies were included from across the globe resulting in thirteen from Europe, fifteen from the USA, five from Middle Eastern countries, four from South American countries, two from Australia, two from South East Asia and two from Canada. The numbers of participants in the cross-sectional studies ranged from 114 to 7557 and included girls and boys at a specific point in time. The age of subjects ranged from 2 to 19 years; however, most studies covered school-age children and adolescents. Results varied greatly in girls and boys and by age group and by type of dairy product.

Two studies were identified showing an association between dairy product intake and increased levels of obesity or indicators of adiposity (one in boys only), whereas all others showed either a null association or inverse association by sex, age group or type of dairy product. In order to explore for reasons for the different results, we looked for themes which might offer an explanation, including type of measurements used, age, race, location, classification of milk and dairy product used, type of dairy product, difference in diets, methodological quality of the study including data analysis, and whether the authors had adjusted for energy and other confounders. We could not identify any consistent reasons for the different results between studies, but detail a summary description of the two cross-sectional studies which found that dairy food intake was associated with increased obesity or indicators of adiposity below, for completeness.

Nezami *et al.* ^(^ ^29^ ^)^ assessed whether dairy product consumption was associated with anthropometric indicators of health in 536 subjects (231 males and 305 females) with a mean age of 14·99 years in a culturally diverse population (37 % Caucasian, 15 % Hispanic, 13 % Asian/Pacific Islander, 10 % African/African American, and 25 % with mixed or other ethnicities). Total dairy product (milk, cheese and sweetened dairy products) consumption was positively and significantly associated with fat mass, fat-free mass and waist:height ratio in males but not in females. Cheese intake was significantly higher in boys compared with girls (2·1 (sd 1·3) servings and 1·7 (sd 1·1) servings, respectively). Normal-weight males consumed more milk than their overweight or obese counterparts but less dairy food in total. The authors adjusted their analysis for age, sex, ethnicity, education of mother, and energy intake, soda intake, physical activity, and milk substitutes intake.

Wiley^(^ ^30^ ^)^ analysed National Health and Nutrition Examination Survey data from 1999 to 2004 and suggested that a dietary pattern characterised by greater milk consumption was associated with increased BMI among children aged 2–10 years, and dairy product intake among 2- to 4-year-old children. The sample included 1493 participants, of which 68 % were Caucasian, 15 % Hispanic and 15 % African/African American. The authors adjusted their analysis for age, birth weight and ethnicity.

We understand that the associations between the exposure (milk and other dairy product consumption) and outcome (body weight/composition) may reflect reverse causality, which means that the presence of adiposity in children may affect their dairy product consumption habits. Contrary to cross-sectional studies, longitudinal analysis of prospective cohort studies represents a more robust design that allows the study of determinants (in this case, milk and other dairy products) with changes over time in measures of adiposity.

## Evidence from longitudinal studies (*n* 31) of the associations between dairy product intake and body weight in children and adolescents

A total of thirty-one studies were identified for review; the size of the cohorts ranged from forty-five to 12 829 and the duration of the follow-up from 8 months to 12 years covering all the periods of growth and human development from infancy to adolescence (Table 1). Of the thirty-one studies, seventeen showed no associations between dairy product, milk or Ca consumption and BMI or measures of adiposity. Rangan *et al.* ^(^ ^31^ ^)^ showed null associations between the high and the low quartiles of energy-adjusted dairy product consumption at 18 months and BMI or weight at 8 years (*P*=0·09) in 335 children from the longitudinal Childhood Asthma Prevention Study (CAPS). However, Garden *et al.* ^(^ ^32^ ^)^ using the same CAPS population after adjusting for potential confounders showed that dairy product consumption (compared with protein, meat and fruit consumption) as a percentage of total energy was inversely associated with BMI (*P*=0·04) and waist circumference (*P*=0·09) at 8 years of age, highlighting that lack of adequate control for potential confounding could have led to conflicting results observed in children. Despite the rigorous assessment of diet at 18 months, intake might not be reflective of the habitual food diet over the entire follow-up period. Phillips *et al.* ^(^ ^33^ ^)^ conducted the first longitudinal analysis in which they considered the pattern of change in dairy product consumption and body weight or fatness during adolescence using annual follow-up assessments of body composition and dietary intake over a 10-year period. After adjusting for covariates, results indicated a null association between dairy product consumption and BMI *z* score (*β*=0·017; *P*=0·65) or percentage body fat (*β*=0·18; *P*=0·51) in 178 initially normal-weight preadolescent girls.

### Differences by type of milk and other dairy products

The majority of prospective studies have examined only milk; thus the impact of ‘type’ of dairy products on the associations between dairy product consumption and body composition remains largely unknown.

With respect to type of milk and the effects of added sugar on health, Fayet-Moore^(^ ^34^ ^)^ recently reviewed the literature related to the role of flavoured or sweetened milk in the diet and weight status of children. Although flavoured milk has similar fat and protein content to white milk, it provides approximately 60 kcal (250 kJ) more per serving portion than plain milk due to added sugars^(^ ^35^ ^)^. Two prospective studies have examined the relationship between milk and measures of adiposity, by differentiating between white milk and flavoured milk^(^ ^35^ ^,^ ^36^ ^)^. A beneficial association of white milk with body weight was reported in the Project EAT (Eating Among Teens) by Vanselow *et al.* ^(^ ^36^ ^)^. The authors showed that plain milk and not flavoured milk consumption assessed by FFQ was inversely related to weight gain (self-assessed) over the 5-year follow-up (*P*=0·05 *v. P*=0·31) without displaying a dose–response relationship in 2294 adolescents and independent of their overweight status. A more recent study employed more accurate measures of dietary intake and body composition by using dietary weighed records and dual-energy X-ray absorptiometry in 2270 children at the age of 10 years^(^ ^35^ ^)^. Results showed that the decrease in percentage body fat was less in overweight/obese children who consumed flavoured milk (17 %) than non-consumers of flavoured milk (83 %) (–0·16 (95 % CI –3·8, 3·5) *v.* –3·4 (95 % CI –6·5, –0·42) %; *P*=0·02). However, there was no association between flavoured milk consumption at 10 years and change in percentage body fat from 11 to 13 years in normal-weight children. It is worth noting that this was the only study that has examined whether the subjects’ BMI at baseline could influence the association between dairy product consumption and body weight or abdominal obesity during childhood or adolescence.

Overall, although the body of literature on flavoured milk is limited, according to the review by Fayet-Moore^(^ ^34^ ^)^ consumption of flavoured milk is not related to weight gain or increased body fat among normal-weight children. However, there are conflicting and inconsistent results among overweight and obese children; thus randomised intervention studies are warranted to examine the effect of flavoured milk consumption on BMI and adiposity. Given the popularity of flavoured milk as the preferred dairy product amongst children, controlled studies with better description of exposure and outcome variables are needed.

### Low-fat *v.* full-fat dairy products

Given the scientific interest on the issue of dairy fat and its contribution to the development of obesity due its high energy content^(^ ^37^ ^)^, we explored the data to assess whether there was any evidence of differential associations of low- and full-fat milk with weight status. Five of the six longitudinal studies examined the relationship between low- or full-fat milk and other dairy products on obesity and indicators of adiposity as a primary outcome of their analysis^(^ ^38^ ^–^ ^43^ ^)^. Although evidence is limited, data suggest that replacing whole milk with reduced-fat milk does not lead to an inverse or positive association between milk and weight gain or prevention of overweight in early childhood. For instance, in 10- to 13-year-olds, Noel *et al.* ^(^ ^38^ ^)^ showed that there was no significant difference between low-fat or skimmed milk *v.* whole milk at 10 years and change in percentage body fat (*P*>0·09) at 11 and 13 years. In preschool children, Huh *et al.* ^(^ ^39^ ^)^ reported inverse association between whole milk, but not low-fat milk, consumption at age 2 years and BMI *z* score (*β*=–0·09 (95 % CI –0·16, –0·01); *P*=0·02) at age 3 years in 852 children, yet there was no association between either whole or low-fat milk with incidence of overweight at age 3 years. Similarly, Scharf *et al.* ^(^ ^40^ ^)^ showed in a longitudinal analysis examining 10 700 children that milk consumption at age 2 years, whether whole or low-fat, was not related to changes in BMI *z* score at the age of 4 years (*P*=0·6). However, children who were normal weight at baseline and consistently drank 1 % fat or skimmed milk at both 2 and 4 years of age had an increased risk of becoming overweight or obese during this time interval (OR 1·57 (95 % CI 1·03, 2·42); *P*<0·05). This contradicts the current American Academy of Pediatrics recommendation in favour of low-fat milk consumption at children ≥2 years^(^ ^44^ ^)^, yet the results could be related to residual confounders that have not been assessed in their analysis such as other food items, total energy intake and physical activity. Berkey *et al.* ^(^ ^41^ ^)^ evaluated the association between annual milk consumption and BMI changes and found that in 12 829 children aged 9–14 years, those drinking ≥3 servings of milk per d had a significantly higher increase in BMI over 4 years, compared with those drinking >1 and ≤2 servings per d. Particularly, high consumption of 1 % milk for boys and skimmed milk for girls had more consistent positive associations with annual BMI gain than did whole or 2 % milk (*P*<0·05). However, adjustment for multiple variables revealed that total energy intake mediated those associations, contributing to the heavier weight status.

### Can dietary calcium reduce body weight?

As dairy products represent the major source of dietary Ca, a few studies reported on the association between dietary Ca and adiposity, without a clear distinction between dairy and non-dairy Ca sources. Nevertheless, as far as Ca is concerned, three studies suggested an inverse relationship between dietary Ca consumption and prevalence of obesity and adiposity in children^(^ ^45^ ^–^ ^47^ ^)^. Skinner *et al.* ^(^ ^46^ ^)^ suggested that increasing the Ca intake with 240 ml skimmed milk or yoghurt daily would reduce children’s body fat by 0·4 %, which in the longer term could reduce the risk of obesity in later childhood, adolescence or adulthood. However, several studies, conducted mostly in girls, showed that dietary Ca over the years was not associated with BMI *z* scores, percentage body fat or trunk fat during adolescence^(^ ^33^ ^,^ ^48^ ^–^ ^50^ ^)^. Very few studies have evaluated the relationship of dietary Ca and adiposity according to sex or ethnicity^(^ ^45^ ^,^ ^47^ ^,^ ^51^ ^)^ and differences in terms of inherent sex characteristics, growth, pubertal status and sexual maturation^(^ ^52^ ^)^ add complexity to the impact of sex on Ca–adiposity associations.

### Differences by age of child

Hasnain *et al.* ^(^ ^53^ ^)^ conducted the longest study using data from the Framingham Children’s Study, in which 103 boys and girls aged 3–5 years old were enrolled and followed annually for 12 years. Prospective analysis between milk and percentage body fat (dual-energy X-ray absorptiometry) indicated that children in the highest tertile of milk consumption (411 ml/d) had 7·3 % lower percentage body fat compared with the lowest (115 ml/d; 30 *v.* 22·6 %; *P*=0·0095) at ages 15–17 years. However, DeBoer *et al.* ^(^ ^54^ ^)^ recently found that in 4-year-old children, drinking ≥4 servings of milk daily had a higher risk of becoming overweight compared with those with an intake of <1 serving daily at age 4 years (OR 1·159 (95 % CI 1·02, 1·32); *P*=0·021), yet the positive association was no longer present at 5 years (OR 1·094 (95 % CI 0·92, 1·31); *P*=0·318). As the authors suggested, this minor adverse association of excess milk consumption with weight status could be age-dependent, with more pronounced association of milk consumption with height over time contrary to its association with weight gain.

### Discussion of longitudinal studies

As summarised in Table 1, there are considerable differences in the exposure and outcome variables and population characteristics, with seventeen studies examining specifically the association between milk consumption and indicators of body weight or adiposity. Data from most prospective studies included in the present review showed that there is a null association between increased milk (*n* 9) or dairy product (*n* 5) consumption and measures of adiposity or body weight. However, there was high variation on the definition and inclusion of dairy foods and type of milks in the studies. For instance, considering the type of milk or fat content, a few studies followed the United States Department of Agriculture definition^(^ ^15^ ^)^ which includes white milk (cow, sheep or goat), while other studies also included sweetened and flavoured, evaporated, condensed, formula, reduced-fat, full-fat or non-fat milk. Similarly, there were notable differences among the seven studies^(^ ^31^ ^–^ ^33^ ^,^ ^39^ ^,^ ^42^ ^,^ ^47^ ^,^ ^55^ ^)^ that investigated total dairy products, with most including any type of yoghurt or cultured milk drinks and natural or processed cheese and some others including dairy desserts such as ice-cream, milkshakes, custard creams and puddings in addition to milk, yoghurt and cheese. However, there were no differences in adiposity outcomes between the studies that included dairy desserts and those that did not. Furthermore, four of them^(^ ^32^ ^,^ ^33^ ^,^ ^39^ ^,^ ^42^ ^)^ reported the portion or percentage energy from milk or other type of dairy products. Based on their reports, milk contributed approximately 50–70 % of the total dairy product consumption followed by yoghurt and cheese, particularly in the highest quartiles or groups of dairy product consumption. Although milk, cheese and yoghurt share several functional properties, differences in their absorption kinetics, macro- and micronutrient content and level of bioactive components could lead to different metabolic effects^(^ ^56^ ^)^. Thus, disaggregating the effects of individual types of dairy product consumption from general dairy products in large-scale epidemiological studies seems prudent.

Few longitudinal studies directly compared body weight or adiposity outcomes related to reduced-fat *v.* full-fat milk or dairy products and results suggest no significant differences between reduced-fat compared with full-fat milk or total dairy products and body weight or adiposity measures^(^ ^33^ ^,^ ^38^ ^–^ ^43^ ^)^. Most of these studies were conducted in the US population and results could have been influenced by reporting bias or residual confounding due to the cultural perception related to fat consumption or other unmeasured associated dietary and health behaviours^(^ ^57^ ^,^ ^58^ ^)^. Additionally, the range or form that dairy food is consumed might differ, given that in the USA, dairy fat is usually consumed as ice-cream or dairy desserts, whilst in Europe plain cheeses or unsweetened yoghurt are the major source of full-fat dairy products^(^ ^37^ ^)^. However, given the limited data, it makes it difficult to draw firm conclusions about whether reduced-fat or full-fat milk and other dairy product consumption may be more beneficial.

Most of the observational studies assessed milk and other dairy product consumption by FFQ, which, compared with food records^(^ ^59^ ^)^, are not the most accurate dietary assessment tool for certain foods, particularly foods that are perceived as unhealthy such as whole milk and cream^(^ ^60^ ^)^. Another acknowledged source of bias in any nutrition research is the over- and under-reporting of dietary intakes, which could lead to gross misclassification of full-fat dairy product intake, particularly among overweight adolescents^(^ ^61^ ^)^. The accuracy of energy reporting may decline as children become older^(^ ^62^ ^)^; thus unbiased estimates of intake are more likely to occur when the dietary reporting is conducted by parents in early childhood (before the age of 6 years)^(^ ^55^ ^)^. Although there is some evidence that milk consumption in childhood tracks over time^(^ ^55^ ^,^ ^63^ ^,^ ^64^ ^)^, a limitation within the longitudinal studies reviewed in the present study is the lack of regular assessment of dairy product and dietary intake throughout childhood and adolescence. The patterns with regard to the type of milk and other dairy product consumption might not be stable over time, especially with the introduction and higher availability of reduced-fat dairy products over the last 25 years.

In addition to diversity in describing the type or fat content of milk and other dairy products, there was variation on the definition and reporting of dairy food serving size, with some studies using the term ‘serves per day’, ‘portions per day’, ‘grams per day’ or ‘times per day’. Similarly, considerable variation was found in the reporting of outcome variables related to weight status and adiposity measures, making it difficult to compare research findings. Future studies in this research area should include a set of well-defined exposure and outcome variables, measured ideally by health professionals to minimise error and reported in a consistent format. For instance, milk, cheese and yoghurt and the type of those dairy foods and fat content reported in g per d as an absolute serving size should be defined. Change in weight in children and adolescents should be reported as BMI *z* score, body weight, percentage body fat, percentage lean body mass and waist circumference.

Adjustment for important confounding factors such as energy intake, diet quality, physical activity levels, baseline BMI, sex, race and socio-economic status were inconsistent and varied among the studies, making it difficult to interpret and compare the results across study cohorts. For instance, twenty-six of the thirty-one studies reviewed included data on both boys and girls, and only sixteen adjusted for sex. Similarly, five^(^ ^35^ ^,^ ^38^ ^,^ ^41^ ^,^ ^42^ ^,^ ^48^ ^)^ of the fifteen studies, which analysed data in school-age children and adolescents, adjusted for pubertal growth development. This is important given that children and adolescents’ body size and composition are influenced by the sex and pubertal growth rate, for example, change in height^(^ ^65^ ^)^, with notable differences in growth rate in the period from early childhood to adolescence. Thus, assumptions that the body composition measures would be independent of pubertal growth stage are invalid and both observational and controlled randomised trials need to consider the influence of pubertal development on the effect of milk and other dairy product consumption on indicators of adiposity. Furthermore, adjustment for energy intake needs to be carefully considered given the high degree of association between increased energy intake and adiposity^(^ ^66^ ^)^. As Berkey *et al.* ^(^ ^41^ ^)^ highlighted, the positive association between milk and annual BMI gain was mediated by the total energy intake and not necessarily by the dairy products *per se*. Given that dairy products could influence body weight through mediating appetite, energy intake and therefore energy regulation as described in the mechanistic section below, analyses should be performed with and without energy intake in the adjustment models. Energy intake was reported in thirteen studies and five studies reported food groups or beverages or macronutrients as indicators of dietary intake in their adjustment models. Thus, inclusion and appropriate control of potential confounders is essential in analyses of all studies.

Sample size and duration varied significantly among the studies, with studies with a protective association between milk and other dairy products and obesity having a smaller sample size (nineteen studies *n*<1000, of which eight showed inverse association) relative to the studies reported an adverse association between increased milk and dairy product consumption and obesity (twelve studies *n*>1000, of which two showed an adverse association). One-third of the studies were funded by the dairy or private industry and five studies showed results favourable for industry compared with the four out of twenty-one public-funded studies, which demonstrated a favourable effect for dairy foods and obesity risk. It is also worth noting that twenty-six out of the thirty-one studies were performed in English-speaking nations (USA (*n* 16), Australia (*n* 4), UK (*n* 4) and Canada (*n* 2)); thus results might not be generalisable to all developed and developing countries.

Overall, the majority of prospective studies showed a null relationship between increased dairy product consumption and measures of adiposity or body weight in children and adolescents with high relative to low or no dairy product consumption. Although data are limited, they suggest that replacing whole milk with reduced-fat milk does not lead to an inverse or positive association between milk and weight gain or prevention of overweight in early childhood.

## Evidence from intervention studies (*n* 20) of the effect of milk and other dairy product intake, with or without energy restriction, on body composition in children

Human intervention studies represent the most robust source of evidence to explore ‘cause-and-effect’ relationships between dairy product consumption and measures of adiposity. Twenty randomised intervention studies examined the effect of dairy products, milk or Ca-rich diets on body weight, body composition and other measures of adiposity during childhood and adolescence. Of the twenty studies identified, ten had bone health as the primary endpoint, but body composition was accurately measured using a state-of-the art method of dual-energy X-ray absorptiometry in all of these studies. However, some of those studies may lack the statistical power needed to detect significant differences in body weight and body composition and therefore should be interpreted with caution. The characteristics and main outcomes of the twenty studies that investigated the effect of milk and other dairy product consumption on indicators of body weight and adiposity are displayed in chronological order in Table 2.

### What do studies that examined bone health reveal about body weight?

The majority of the studies that were primarily designed to examine bone health failed to detect differences among the treatments^(^ ^67^ ^–^ ^74^ ^)^. For example, Chan *et al.* ^(^ ^67^ ^)^ conducted a 12-month randomised parallel study in pubertal girls and found no differences in body weight, lean body mass and fat mass between the high dairy Ca group (1200 mg Ca/d) and the control group (usual diet). Likewise, Cadogan *et al.* ^(^ ^68^ ^)^ reported no effect of whole or reduced-fat milk consumption (568 ml/d) on body composition in 12-year-old girls for 18 months, and Merrilees *et al.* ^(^ ^69^ ^)^ found no effect with the provision of 1000 mg Ca/d from dairy foods over 2 years on body weight, body lean mass and fat mass in post-pubertal teenage girls compared with those girls on their usual diet. Similar neutral effects were observed in an 18-month randomised parallel study in 9- to 10-year-old Chinese children, between the 80 g (equivalent to 250 ml whole milk) or 40 g milk powder supplementation group and the control group (usual diet)^(^ ^72^ ^)^. A more recent 18-month randomised parallel study showed that 240 healthy-weight and overweight 8- to 16-year-old boys and girls when assigned to either receive three servings of dairy products/d (1500 mg Ca/d) or maintain their usual diet (1000 mg Ca/d) had no differences in body weight and fat gain^(^ ^74^ ^)^. In contrast, one study conducted in 757 Chinese girls with low habitual Ca diet and high prevalence of vitamin D deficiency showed weight gain and enhanced growth^(^ ^75^ ^)^. Du *et al.* ^(^ ^75^ ^)^ conducted the largest school-milk intervention trial, where the 10-year-old girls were randomly assigned to a group that received 330 ml of Ca-fortified milk, a group that received 330 ml of Ca-fortified milk plus vitamin D or a control group (habitual diet). There were no differences in body height and weight between the three groups over the 24-month study period, yet milk consumption substantially increased height, sitting height and body weight when expressed by mean percentage changes from baseline compared with the control.

### Dairy products *v.* dietary/supplemental calcium

Although the effect of exclusively supplemental Ca on body composition is out of the scope of the present review, it is worth mentioning the only study that was designed to examine the effects of both dairy products and Ca supplements (calcium carbonate tablets) on bone mass accrual and body composition, therefore allowing a direct comparison and an important insight into the purported bioactive components of dairy products^(^ ^71^ ^)^. In this 2-year randomised trial in 10- to 12-year-old girls, findings indicated no differences in body weight, height, lean tissue and fat mass between the cheese (1000 mg/d), the supplemental Ca (1000 mg/d), the placebo and the reference group (habitual high Ca intake >900 mg/d). The authors suggested that the lack of differences in body composition could have been masked by the diverse growth pattern in the girls. In concordance with the findings based on bone health studies, Lappe *et al.* ^(^ ^76^ ^)^ showed that 9-year-old girls in the high Ca diet group (1656 (sd 191) mg Ca/d) compared with the control group (usual diet: 961 (sd 191) mg Ca/d) gained similar body weight (34 *v.* 33 %; *P*>0·05), lean mass (31 *v.* 31 %; *P*>0·05) and fat mass (38 *v.* 33 %; *P*>0·05) despite the nearly two-fold increase in dairy product consumption in the treatment group over 2 years. Based on a recent meta-analysis of combined results from nine studies including both observational and randomised clinical trials^(^ ^77^ ^)^, Ca intake (derived from Ca-rich foods, dairy products and Ca supplements) was inversely related to body weight gain in children and adolescents (mean –0·26 (95 % CI –0·41,–0·11) kg; *P*<0·001).

### Differences by type of milk and other dairy products

Most intervention trials, like observational studies, have examined only milk or total dairy products, with no studies having addressed the effect of ‘type’ of dairy products on childhood and adolescent obesity. Arnberg *et al.* ^(^ ^78^ ^)^ conducted a 3-month randomised parallel study (the Milk Components and Metabolic Syndrome (MoMS) study) in overweight subjects aged 12–15 years and showed that adolescents in the skimmed milk (1 litre/d), whey- and casein-based drink (35 g protein/l per d) supplementation groups increased BMI-for-age *z* scores compared with the control group (water intake) and pre-test control subgroup (3 months before intervention). However, given that puberty is a period of rapid growth, BMI is not the most representative and adequate index of body composition. Thus, Larnkjaer *et al.* ^(^ ^79^ ^)^ in a follow-up analysis of this study demonstrated that milk-based drinks increased both body fat and lean mass. Although milk-based drinks improved body composition by increasing lean mass and maintaining fat mass relative to pre-test control, they had a less favourable effect on body composition than water intake. However, this could be attributable to the replacement of one energy drink for a non-energy beverage such as water. Replacement of one energy drink for another was examined in a 16-week study, which included an intervention with increasing flavoured milk, whilst almost eliminating sugar-sweetened beverage consumption by delivering milk beverages to the homes of overweight and obese 8- to 10-year-old children^(^ ^80^ ^)^. Although no differences in percentage of body fat were observed, milk consumption increased the accretion of lean body mass compared with the control (usual diet). Of all the randomised intervention studies reviewed, the recent study by Lappe *et al*.^(^ ^81^ ^)^ is the first study to report the effects of dairy food on body weight and fat in adolescents. They enrolled 13- to 14-year-old girls with BMI between the 50th to 98th percentiles and with habitual low Ca intake (<600 mg/d) and assigned them to either the intervention dairy food group (reduced-fat milk and yoghurt, ≥1200 mg Ca/d) or the control group (usual diet) for 12 months. There were no differences in weight and fat gain between the dairy food and control groups. In an explanatory analysis, they found that the effect of reduced-fat milk did not differ from yoghurt on change in percentage of body fat or weight, yet due to the nature of the intervention incorporating both dairy products, the effect of each type of dairy product remains to be explored.

### Interventions aimed to manage childhood overweight and obesity, which included advice on different milk and other dairy products

Two studies included healthy eating counselling as part of their weight management intervention^(^ ^82^ ^,^ ^83^ ^)^. Findings from a 16-week randomised parallel trial in 8- to 9-year-old overweight children, indicated that high milk (4 × 236 ml/d) compared with low milk (1×236 ml/d) consumption tended to decrease visceral adipose tissue (–0·18 (sem 0·08) *v.* –0·11 (sem 0·05) litres; *P*>0·05), despite the fact that there was no significant change in body weight or body composition^(^ ^82^ ^)^. As authors suggested, a larger sample size and a longer duration might have been needed to adequately examine and detect body composition changes between the milk groups. Furthermore, a randomised controlled trial in 120 obese and prepubescent children examined the effect of a dairy product-rich diet on long-term weight management^(^ ^83^ ^)^. The children were randomly assigned to a dairy product-rich diet group (>800 mgCa/d, without any energy restriction), energy-restricted diet or control group and all of them had six healthy lifestyle family sessions once per month during the 6-month intervention. BMI standard deviation scores and waist circumference were decreased significantly in all groups after the 6 months. A follow-up was done twice yearly for 3 years and a significant increase was observed in BMI and waist circumference given the period of rapid growth. However, the increase was significantly lower in the dairy product group compared with the other two groups suggesting that in addition to lifestyle modifications, a dairy food-rich diet could be a potent strategy for weight management in young overweight children.

Similarly, a recent 38-month study in 7- to 10-year-old children^(^ ^73^ ^)^ included a behavioural modification and nutritional education intervention to gradually increase dietary Ca intake to 1500 mg/d compared with the usual-care group (a single 45 min nutrition session). Results showed an increase in Ca intake in the intervention group and an inverse association between dietary Ca intake and gain in BMI and fat mass irrespective of treatment group.

Although several studies have examined the effect of milk or dairy product consumption on alterations in body weight and composition under energy restriction in adults^(^ ^84^ ^,^ ^85^ ^)^, there is only one study which explored the relationship between dairy product consumption and BMI or fat mass in overweight and obese children during energy restriction^(^ ^86^ ^)^. In this 12-week study, 120 overweight and obese children on an energy-restricted regimen (500 kcal/d (2090 kJ/d) below requirement) were assigned to two, three or four servings of dairy products/d. There were significant reductions in BMI, body weight and body fat, yet these reductions were due to the energy deficit and independent of the effect of increasing dairy product consumption.

### Discussion of intervention studies

Examining Table 2, many aspects of the study methodology used in the included studies differed considerably, notably the length of the intervention (ranging from 3 months to 2 years) and the number of participants (for example, twenty-eight to 757), with half of the studies using dairy products and the other half milk consumption as part of their intervention. Data from the majority of the studies reviewed show that there is a neutral effect of dairy product (*n* 10) and milk (*n* 9) consumption on body weight and body composition in children and adolescents. More specifically, all the studies that examined the impact of a dairy food-rich diet relative to habitual diet on indicators of adiposity showed neutral effect and three studies^(^ ^75^ ^,^ ^78^ ^,^ ^79^ ^)^ that used milk consumption as the intervention showed an increase in body weight. However, as pointed out in the description of those studies, the increase in body weight was accompanied by an increase in lean mass^(^ ^78^ ^,^ ^79^ ^)^ or overall growth^(^ ^75^ ^)^. Specifically, in the study by Du *et al.* ^(^ ^75^ ^)^, as the largest school-milk intervention trial, the 757 included girls were healthy but on a persistently low-Ca diet and had a high prevalence of vitamin D deficiency^(^ ^87^ ^)^, thus representing a population that is not comparable with most of the intervention studies examined.

It is also noteworthy that there are no studies with body composition or any other indicators of obesity that have included intervention with both regular or reduced-fat milk or other type of dairy products, thereby not allowing a direct comparison. The processing of dairy foods often involves removal of whey protein from cheese, or addition of ingredients such as sugar in flavoured milk, yoghurt and ice-creams^(^ ^88^ ^)^. It can also lead to a number of alterations or biochemical changes in milk constituents, macronutrients and bioactive factors through such as insulin-like growth factor-1 (IGF-1)^(^ ^89^ ^)^. Thus, using total dairy product consumption might not be a suitable variable to elucidate the underlying impact of milk and other type of dairy products on adiposity indicators. Among the randomised controlled trials reviewed that included total dairy products, most included milk, cheese and yoghurt with a few studies including additionally dairy desserts^(^ ^69^ ^)^ or any curd products^(^ ^83^ ^)^. However, there were no reports in the percentage of milk or other dairy products contribution to the total dairy product consumption, which does not allow examination of any differential effects. Thus, disaggregation of dairy products, such as reduced-fat compared with full-fat milk, fermented products such as yoghurt and cheese and in general those that are encouraged by the dietary guidelines as opposed to those that should be consumed sporadically (i.e. ice-cream, milkshakes), is essential in the analysis of children and adolescent obesity.

Dairy food serving sizes were adequately defined in most of the intervention studies with feasible quantification of milk and total dairy product consumption with the observed outcomes. However, variation in the definition of serving size/portions and high, low or habitual milk or dairy product consumption among the studies is apparent, which makes it very difficult, if not inappropriate, to compare results across studies conducted in different location and populations. For instance, while in Finland a habitual diet contains approximately 900 mg Ca/d^(^ ^71^ ^)^, in the USA population this is reported to contain between 600 and 700 mg Ca/d^(^ ^74^ ^,^ ^81^ ^,^ ^90^ ^)^. There were also variations in the way the outcome variable is reported within studies, with most studies reporting body weight, BMI, lean body mass, percentage body fat while very few reported BMI *z* score, waist circumference or waist:height ratio, which have been suggested as more appropriate for children and adolescents and important early markers for the development of chronic diseases^(^ ^91^ ^,^ ^92^ ^)^.

The majority of the included studies were predominately conducted in girls (*n* 7) and in school-aged children and adolescents. There were no differences in the outcome variables related to sex comparing the studies that included only girls and those that included both sexes (*n* 12). However, sex was adjusted in seven out of twelve studies and rate of growth (pubertal or Tanner stage) adjusted in only four^(^ ^72^ ^,^ ^74^ ^,^ ^78^ ^,^ ^79^ ^)^ out of eighteen studies conducted in school-aged children and adolescents (age 8–18 years).

It is important to recognise that many of the intervention studies (thirteen out of twenty) included had a non-intervention control group, reported as usual or habitual diet often low in milk or dairy products, as the comparator to increased milk and total dairy food intervention group. Although this information allows separating the effects of treatment group on measures of adiposity, it does not provide insight on whether the effects observed are attributable to the increased milk or dairy product consumption *per se*, or to the concomitant manipulation of other food groups. Only one study utilised an isoenergetic replacement food such as fruit juice^(^ ^70^ ^)^ as a comparator to milk and another study increased the consumption of milk by decreasing the consumption of sugar-sweetened beverages^(^ ^80^ ^)^, therefore separating the effect of milk. Both of those studies showed a neutral effect of increased milk consumption compared with fruit juice or usual diet on lean and fat mass^(^ ^70^ ^,^ ^80^ ^)^. Future studies should try to include both positive (i.e. isoenergetic food or beverages) and negative comparators (i.e. water or no milk group) to increase the ability to draw robust conclusions with regard to the treatment effect.

Although intervention studies are highly diverse considering the duration, location or sample size (Table 2), there are no clear patterns or trends identified between the interventions of milk or other dairy products that influence body weight and indicators of adiposity positively, neutrally or negatively. Additionally, half of the intervention studies were funded by the dairy or food industry and results showed that the source of funding did not affect the results.

Taken together, data from the majority of the intervention studies reviewed show that there is a neutral effect of dairy product and mainly milk consumption compared with the habitual diet on body weight and body composition in children and adolescents. Inclusion of milk and other dairy products as part of an energy-restricted diet did not enhance, but neither did it adversely affect, BMI or weight loss. However, given the limited data available from studies conducted under conditions of energy restriction, no conclusions can be drawn.

## Plausible mechanisms underlying the effect of dairy components on body-weight regulation

Given the inconsistencies among the studies reviewed, there is a great need to understand the mechanisms by which dairy products affect energy balance and consequently body weight or composition. As reviewed elsewhere^(^ ^93^ ^–^ ^95^ ^)^, a number of different plausible mechanisms have been suggested, with the most frequently cited mechanism relating to the effects of intracellular ionised Ca on adipocyte metabolism^(^ ^96^ ^,^ ^97^ ^)^. According to this theory, an increase in dietary Ca via its influence on circulating calcitropic hormones (suppression of the 1,25-hydroxyvitamin D and parathyroid hormone concentrations) reduces the concentration of intracellular ionised Ca in human adipocytes. The resultant decrease in ionised Ca stimulates lipolysis, inhibits *de novo* lipogenesis and increases fat oxidation^(^ ^98^ ^,^ ^99^ ^)^. Gonzalez *et al.* ^(^ ^100^ ^)^ showed in a meta-analysis that an increase in dietary Ca by 800 mg/d predicted an 11 % increase in fat oxidation with more pronounced effects when habitual chronic Ca intake was low (<700 mg/d). However, the hypothesis of reduced adipogenesis through dietary Ca has been refuted by some human studies^(^ ^101^ ^,^ ^102^ ^)^. There is also some evidence that Ca in the form of dairy foods exerts greater effect than Ca supplements^(^ ^103^ ^)^. This could be attributable to the synergistic actions of Ca with several bioactive compounds present in dairy products such as branched-chain amino acids, i.e. leucine^(^ ^104^ ^)^. Peptides in whey protein inhibit angiotensin-converting enzyme, which suppresses angiotensin II hormone production and consequently stimulation of adipocyte lipogenesis, resulting in reduction of fat accumulation^(^ ^105^ ^,^ ^106^ ^)^. Fatty acids in dairy products such as medium-chain fatty acids and conjugated linoleic acid have also been shown to reduce lipogenesis and increase fat oxidation in the adipocyte via suppressing the expression of pro-adipogenic cell signals including PPARγ^(^ ^107^ ^–^ ^110^ ^)^.

Additionally, dietary Ca may exert the anti-obesity effect mediated by increased faecal fat excretion^(^ ^111^ ^,^ ^112^ ^)^. Specifically, Ca interferes with fat absorption in the gastrointestinal tract by binding fatty acids to form insoluble Ca soaps or by creating precipitates with phosphate and bile acids, which decreases the digestible energy from the diet and contributes to a negative energy balance^(^ ^113^ ^)^. Christensen *et al*.^(^ ^114^ ^)^ showed in a meta-analysis of fifteen studies, including one study in children^(^ ^115^ ^)^, that increased Ca intake from both supplements and dairy products of 1241 mg/d increased faecal fat excretion by 5·2 g/d. However, limited studies have examined these mechanisms in children and adolescents^(^ ^81^ ^,^ ^90^ ^)^ and results do not support any effect of dietary Ca or dairy products on energy, fat or N balances in overweight adolescents when energy intake and physical activity are controlled. These results were independent of the Ca source (dairy foods or calcium carbonate supplements) or when stratified by the baseline BMI percentile. As the authors stated, the contrasting results between adolescents and adults regarding faecal fat excretion could be attributable to the higher faecal Ca in adults compared with adolescents who had similar Ca intake^(^ ^90^ ^)^. Furthermore, in addition to sex-related differences (higher visceral fat deposition^(^ ^116^ ^)^ and thermogenic oxidation^(^ ^90^ ^)^ in boys), several dynamic metabolic changes occur in a period of rapid growth and puberty that could further complicate the mechanistic pathways^(^ ^117^ ^)^.

Another proposed mechanism refers to the Ca appetite concept, which according to Tordoff^(^ ^118^ ^)^, dietary Ca depletion triggers the appetite for Ca-rich foods. There are some plausible mechanisms, which rely on the identification of certain genes responsible for taste recognition^(^ ^119^ ^)^ and an increase in the fasting anorexigenic hormone leptin^(^ ^120^ ^)^ or postprandial levels of anorexigenic glucose-dependent insulinotropic peptide and glucagon-like peptide after ingestion of Ca^(^ ^121^ ^)^, yet with inconsistent results^(^ ^122^ ^,^ ^123^ ^)^. Specific components of dairy foods and mainly whey and casein proteins have been associated with increased satiety^(^ ^124^ ^)^ through delayed gastric emptying^(^ ^125^ ^)^, and regulation of the concentration of plasma amino acid^(^ ^126^ ^)^ and gastrointestinal hormones such as cholecystokinin^(^ ^127^ ^)^, peptide YY^(^ ^128^ ^)^ and gastrin^(^ ^129^ ^)^. However, it remains unknown whether the satiating effect is due to Ca or the dairy matrix^(^ ^130^ ^)^, with inconsistent results in the limited studies conducted in children and adolescents, as discussed later.

Certain dairy products such as yoghurt might exert the anti-obesity effect mediated by manipulation of the composition and metabolic activity of gut microbiota^(^ ^131^ ^,^ ^132^ ^)^. The composition of the intestinal microbiota differs between normal-weight and obese/overweight children^(^ ^133^ ^,^ ^134^ ^)^ and there is evidence that early differences in gut microbiota composition in children could predict overweight^(^ ^135^ ^)^. Given that fermented dairy products are a source of probiotics, some plausible mechanisms, which are still poorly understood, suggest an interaction of probiotics with indigenous bacteria in the gastrointestinal tract that might influence the metabolic pathways involved in lipid metabolism^(^ ^136^ ^)^. Although the few studies conducted in that area showed beneficial effects of probiotics on weight management in children^(^ ^137^ ^,^ ^138^ ^)^, further research is clearly needed.

A few observational and intervention studies showed a positive association between milk and other dairy products^(^ ^41^ ^,^ ^43^ ^,^ ^75^ ^,^ ^78^ ^,^ ^79^ ^)^ and particularly dairy proteins^(^ ^139^ ^,^ ^140^ ^)^ with weight gain. Putative mechanisms explaining increased weight gain include the stimulation of IGF-1^(^ ^141^ ^–^ ^143^ ^)^, which has a negative impact on preadipocyte differentiation and multiplication^(^ ^144^ ^,^ ^145^ ^)^. However, there are conflicting results with regards to the relationship between IGF-1 concentration and BMI in children^(^ ^146^ ^–^ ^148^ ^)^.

In addition, milk proteins have also been shown to stimulate insulin secretion^(^ ^149^ ^–^ ^151^ ^)^, which is another growth hormone due to its binding to IGF-1 receptors, thus promoting cell replication in connective and musculoskeletal tissue^(^ ^152^ ^)^. Further research is needed in order to elucidate the putative mechanisms underlying the impact of dairy components on body weight regulation.

### Evidence from intervention studies of the effect of dairy product intake on appetite and energy intake regulation

Given that obesity is characterised by an imbalance between energy intake and expenditure, understanding the factors or dietary components that could regulate appetite and energy intake has been a substantial focus in recent years. Although several studies have examined the effect of milk and other dairy product consumption as whole foods on appetite and subsequent meal energy intake in adults^(^ ^153^ ^–^ ^156^ ^)^, there is a limited number of studies conducted in children^(^ ^157^ ^–^ ^161^ ^)^. Brindal *et al.* ^(^ ^157^ ^)^ showed that there was no difference in subsequent appetite or energy intake at the *ad libitum* lunch 3 h after the consumption of a full milk beverage compared with isoenergetic half milk/glucose and glucose beverages in 10- to 12-year-old children. In contrast, Mehrabani *et al.* ^(^ ^158^ ^)^ recently showed that 10- to 12-year-old obese boys who consumed a fixed content breakfast with low-fat milk for two consecutive days had lower subjective appetite ratings and subsequent energy intake at lunch 5 h after, compared with apple juice and water (1010 kcal (4226 kJ), 1059 kcal (4431 kJ) and 1236 kcal (5171 kJ), respectively; *P*<0·001). Furthermore, Green *et al.* ^(^ ^161^ ^)^ showed that acute mid-morning milk consumption as a snack reduced *ad libitum* energy intake compared with an isoenergetic and isovolumetric serving of fruit juice in adolescent boys. Differences in subjects’ characteristics (obese boys *v.* normal-weight boys and girls) and time interval between breakfast or snack and the *ad libitum* lunch could explain the discrepancy in results between the studies. Furthermore, Mehrabani *et al.* ^(^ ^158^ ^)^ provided one serving of fruit between the breakfast and lunch which could have assisted in prolonging the feelings of satisfaction up to 5 h, whilst Brindal *et al.* ^(^ ^157^ ^)^ used an interval of 3 h where appetite ratings have returned to baseline values. Despite the modest decrease in energy intake by 50 kcal (209 kJ) between milk and apple juice, the authors suggested it could help with energy control and maintaining a healthy weight in the longer term.

### Free-living setting outcomes

The majority of the studies have investigated appetite and energy intake as the primary endpoints within a laboratory setting but some of the studies, which included assessment of energy intake as part of body weight measurements, could shed some light on the effect of dairy foods on energy intake in a free-living environment. For instance, Lappe *et al.* ^(^ ^76^ ^)^ showed that girls consuming a Ca-rich diet were not ingesting more energy relative to those on their habitual diet and Albala *et al.* ^(^ ^80^ ^)^ reported decreased energy intake by the concurrent replacement of sugar-sweetened beverages with milk consumption compared with control (–91 *v.* 9·7 kcal/d (–381 *v.* 41 kJ/d); *P*=0·03). Similarly, Andersen *et al.* ^(^ ^162^ ^)^ recently revisited the MoMS intervention study, investigating the effects of increased water or milk-based drinks on total energy intake and dietary patterns in overweight adolescents. Although water intake reduced (–236 kcal/d (–987 kJ/d) while milk-based drinks did not alter total energy intake during the 12-week intervention compared with the background diet (baseline), both water and milk-based drinks had a favourable effect on the diet by decreasing the consumption of convenience foods, including sugar-sweetened beverages. This is particularly important given that there were no other restrictions in the diet and the milk-based drinks were added as extra intake, implying a compensatory effect of those on energy balance and reflecting positive dietary changes in usual life and free-living behaviour. Finally Green *et al.* ^(^ ^161^ ^)^ recently showed no statistically significant differences between chronic (28 d) milk and fruit juice consumption as a mid-morning snack on change in total daily energy intake. However, with the group consuming milk as a mid-morning snack, total daily energy intake declined over the 28 d (*P*=0·013); no such change was observed in the group consuming fruit juice.

### Effect of type of milk or dairy product on satiety and energy intake

Considering the impact of the type of dairy foods with regard to the fat content on satiation and energy intake, Kling *et al.* ^(^ ^159^ ^)^ recently showed that there was a compensatory effect of full-fat relative to low-fat milk consumption when served with lunch on preschoolers’ food intake, although there were no differences in total meal energy intake (lunch + milk). However, there was a sex effect; lunch intake was reduced by 43 (sem 8) kcal (180 (sem 33) kJ) (*P*=0·001) after full-fat milk without altering total meal energy intake for boys, whereas in girls there was no reduction in lunch intake, which increased total meal energy intake by 24 (sem 10) kcal (100 (sem 42) kJ) (*P*=0·03). However, this was an acute study and it remains unknown whether energy compensation persists over the whole day or in intakes over the long term. There is one study which examined the effect of replacing regular-fat with reduced- or low-fat dairy products on dietary intakes and health outcomes^(^ ^163^ ^)^. In this 24-week cluster randomised controlled trial, parents and their 4- to 13-year-old children were randomly assigned to behavioural nutrition education to change to reduced-fat dairy products (intervention group) or reduce screen time (control group). The results indicated that there was a 74 % decrease in regular-fat dairy product intake in the intervention group, which led to a reduction of 3·3 percentage points in saturated fat compared with the control group at week 24. However, there was no difference in total energy intake (–195 (95 % CI –772, 383) kJ; *P*=0·504) or BMI *z* score and waist circumference between the two groups. The authors in a secondary analysis of this trial tried to address whether there was compensation or energy adjustment by incorporating other foods in the diet when lower-energy-dense dairy products were introduced^(^ ^164^ ^)^. The results showed that replacing regular- with reduced-fat dairy foods did not adversely have an impact on children’s overall food intake by revealing no differences in food group contribution to energy intake between the intervention and the control group at week 24. The foods that contained excess fat, added sugar and/or salt had the largest contribution to children’s total energy intake (27 %) and saturated fat intake (33 %), highlighting that reduction of energy-dense foods is necessary to achieve better control of saturated fat and total energy intake.

In general, inclusion of any dairy products into weight-maintenance diets did not lead to a higher overall energy intake, implying a partial compensatory effect of milk on energy balance. Further, adequately designed studies are needed to examine if habitual consumption of milk and other dairy products make an impact on appetite regulation and consequently energy intake in children and adolescents.

## Overall conclusions

This critical and comprehensive review of evidence based on ninety-four studies of different study designs found that milk and other dairy products are consistently not associated, or inversely associated, with body fatness in children relative to low or no dairy product consumption. More specifically, results from observational studies presented as both unadjusted, and as adjusted, for energy intakes indicate that adjustment for energy intake tended to change negative associations to null or neutral. Our findings are based on forty-three cross-sectional and thirty-one longitudinal studies. Only six studies of the seventy-four observational studies reviewed found any positive association between any component of milk or other dairy products and body fatness in children, and for two of these five studies the exposure was milk protein. The majority of the twenty intervention studies showed no differences in body weight and body composition between children and adolescents who increased dairy food and mainly milk consumption and those who maintained their habitual diet. Only one study included energy intake restriction and increased dairy product consumption; neither BMI nor weight loss was enhanced or adversely affected, yet no firm conclusions can be drawn due to limited data available under energy restriction.

We did endeavour to assess whether the relationship was different by type of milk and milk product. Although some studies did report differences, we consistently found that the type of product did not change the overall direction of travel for both observational and intervention studies. We found that there appeared to be very little difference in the results between milks of differing fat content, regardless of study design.

We also endeavoured to assess whether the relationship was different by age of the child. Most studies were conducted in 8- to 12-year-olds, but only a few studies were conducted in preschool children. The relationship between milk and other dairy products, and body fatness in children, did not appear to change during childhood except in the preschool years. Due to the limited data available, and the inconsistency of the results, it is not possible to make any inference about the relationship between milk and other dairy products with obesity and indicators of adiposity, and age of the child. However, we do suggest that further research is warranted on this topic in children < 5 years of age.

Milk and dairy foods are nutrient rich and make a significant contribution to Ca, iodine, riboflavin, vitamin B_12_, K and vitamin A intakes in children. On this basis, we conclude that there is limited and no definitive evidence to support a concern to limit the consumption of milk and other dairy products by children on the grounds that they may promote obesity. Furthermore, the existing evidence shows that there is no accepted underlying mechanistic rationale to support the hypothesis that milk and other dairy products promote excess weight gain, or increase appetite.

Further research is needed to better understand the role of different milks and different dairy foods in childhood obesity. The new and emerging range of products (including plant-based alternatives) being used as dairy milk substitutes has yet to be evaluated in scientific studies.

## Supplementary material

For supplementary material accompanying this paper visit https://doi.org/10.1017/S0954422418000227. click here to view supplementary material
