# Supplementary material for: A critical review of the role of milk and other dairy products in the development of obesity in children and adolescents
Source: Nutr Res Rev. 2018 Nov 27;32(1):106–27. doi: 10.1017/S0954422418000227 (PMC6536827; doi:10.1017/S0954422418000227)
Supplement: Supplementary file 1 [file S0954422418000227sup001.docx]

**Supplementary Table S1.** Examples of milk and dairy products commonly consumed in the British diet^1^

| **Drinking milk** | **Cheese** | **Yoghurt, dairy based desserts & other ingredients** |
| --- | --- | --- |
| Whole milk | Hard cheese (cheddar, red Leicester) | Yoghurt |
| Semi-skimmed milk | Soft cheese | Greek/Greek style yoghurt |
| 1% milk | Ripened cheeses | Fromage frais |
| Skimmed milk | Quark | Cream (clotted, double & single) |
| Lactose free milk | Cottage cheese | Soured cream |
| Skimmed milk powder | Curds | Ice-cream & frozen yoghurt |
| Flavoured milk | Paneer | Custard |
| Cultured milk (e.g. Kefir) | Cream cheese | Milk pudding |
|  |  | Whey/casein powder |

^1^ Data taken from Finglas *et al.*^(^[^17^](#_ENREF_17)^)^ and Public Health England^(^[^175^](#_ENREF_175)^)^. Composition of foods integrated dataset (CoFID)^.^

**Supplementary Table S2.** Nutrient composition per 100g, and per standard adult portion, of milk and dairy products^1^

|  | **Whole milk** | | **Semi-skimmed** | | **Skimmed milk** | | **Cheddar cheese** | | | | **Cottage cheese** | | **Spreadable cheese (Soft white low-fat)** | | **Whole milk yoghurt** | | | | **Low-fat yoghurt** | | | | **Fromage frais** | | | |
| --- | --- | --- | --- | --- | --- | --- | --- | --- | --- | --- | --- | --- | --- | --- | --- | --- | --- | --- | --- | --- | --- | --- | --- | --- | --- | --- |
|  |  |  |  |  |  |  | **Regular** | | **Reduced fat (30% less)** | | **Plain, regular** | |  |  | **Plain** | | **Fruit** | | **Plain** | | **Fruit** | | **Per 100g** | | | |
|  | **per 100g** | **per 200ml glass (206g)** | **per 100g** | **per 200ml glass (206g)** | **per 100g** | **per 200ml glass (208g)** | **Per 100g** | **per 30g serve** | **Per 100g** | **per 30g serve** | **Per 100g** | **per 40g serve** | **Per 100g** | **per 40g serve** | **per 100g** | **per 150g pot** | **per 100g** | **per 150g pot** | **per 100g** | **per 150g pot** | **per 100g** | **per 150g pot** | **Children's fortified - fruit** | **Virtually fat free - plain** | **Virtually fat free - fruit** |  |
| Energy (kcal) | 63 | 130 | 46 | 95 | 34 | 71 | 416 | 125 | 314 | 94 | 103 | 41 | 99 | 40 | 79 | 119 | 109 | 164 | 57 | 86 | 78 | 117 | 99 | 49 | 50 |  |
| Protein (g) | 3.4 | 7.0 | 3.5 | 7.2 | 3.5 | 7.3 | 25.4 | 7.6 | 27.9 | 8.4 | 9.4 | 3.8 | 11.9 | 4.8 | 5.7 | 8.6 | 4.0 | 6.0 | 4.8 | 7.2 | 4.2 | 6.3 | 5.8 | 7.7 | 6.8 |  |
| Carbohydrate (g) | 4.6 | 9.5 | 4.7 | 9.7 | 4.8 | 10.0 | 0.1 | 0.0 | 0.8 | 0.2 | 3.1 | 1.2 | 5 | 2 | 7.8 | 11.7 | 17.7 | 26.6 | 7.8 | 11.7 | 13.7 | 20.6 | 13.2 | 4.6 | 5.6 |  |
| Total sugars (g) | 4.6 | 9.5 | 4.7 | 9.7 | 4.8 | 10.0 | 0.1 | 0.0 | 0.1 | 0.0 | 3.1 | 1.2 | 5 | 2 | 7.8 | 11.7 | 16.6 | 24.9 | 7.5 | 11.3 | 12.7 | 19.1 | 11.8 | 4.4 | 4.9 |  |
| Fat (g) | 3.6 | 7.4 | 1.7 | 3.5 | 0.3 | 0.6 | 34.9 | 10.5 | 22.1 | 6.6 | 6.0 | 2.4 | 3.6 | 1.4 | 3.0 | 4.5 | 3.0 | 4.5 | 1.0 | 1.5 | 1.1 | 1.7 | 2.9 | 0.1 | 0.2 |  |
| of which saturates | 2.3 | 4.7 | 1.1 | 2.3 | 0.1 | 0.2 | 21.7 | 6.5 | 13.8 | 4.1 | 3.2 | 1.3 | 2.4 | 0.96 | 1.9 | 2.9 | 2.0 | 3.0 | 0.7 | 1.1 | 0.8 | 1.2 | 1.9 | 0.1 | 0.1 |  |
| Monosaturates | 1.0 | 2.1 | 0.4 | 0.8 | 0.1 | 0.2 | 9.4 | 2.8 | 6.5 | 2.0 | 1.7 | 0.7 | 0.9 | 0.36 | 0.8 | 1.2 | 0.7 | 1.1 | 0.2 | 0.3 | 0.3 | 0.5 | N | Tr | 0.1 |  |
| Polyunsaturates | 0.1 | 0.2 | Tr | Tr | Tr | Tr | 1.1 | 0.3 | 0.6 | 0.2 | 0.2 | 0.1 | 0.1 | 0.04 | 0.1 | 0.2 | 0.1 | 0.2 | Tr | Tr | Tr | Tr | N | Tr | Tr |  |
| Trans fatty acids | 0.1 | 0.2 | 0.1 | 0.2 | Tr | Tr | 1.4 | 0.4 | N | N | 0.3 | 0.1 | 0.1 | 0.04 | 0.1 | 0.2 | 0.1 | 0.2 | Tr | Tr | Tr | Tr | N | Tr | Tr |  |
| Fibre (g) | 0 | 0 | 0 | 0 | 0 | 0 | 0 | 0 | 0 | 0 | 0 | 0 | 0 | 0 | 0 | 0 | N | Tr | N | N | 0.2 | 0.3 | N | 0 | 0.4 |  |
| Thiamin (mg) | 0.03 | 0.06 | 0.03 | 0.06 | 0.03 | 0.06 | 0.03 | 0.01 | 0.03 | 0.01 | 0.05 | 0.02 | 0.05 | 0.02 | 0.06 | 0.09 | 0.12 | 0.18 | 0.12 | 0.18 | 0.12 | 0.18 | 0.11 | 0.13 | 0.03 |  |
| Riboflavin (mg) | 0.23 | 0.47 | 0.24 | 0.49 | 0.22 | 0.46 | 0.39 | 0.12 | 0.53 | 0.16 | 0.24 | 0.10 | 0.41 | 0.16 | 0.27 | 0.41 | 0.16 | 0.24 | 0.22 | 0.33 | 0.21 | 0.32 | 0.29 | 0.20 | 0.37 |  |
| Niacin (mg) | 0.2 | 0.4 | 0.1 | 0.2 | 0.1 | 0.2 | 0.1 | 0.0 | 0.1 | 0.0 | 0.2 | 0.1 | 0.1 | 0.04 | 0.2 | 0.3 | 0.1 | 0.2 | 0.1 | 0.2 | 0.1 | 0.2 | 0.1 | 0.1 | 0.1 |  |
| Niacin from Tryptophan (mg) | 0.6 | 1.2 | 0.6 | 1.2 | 0.7 | 1.5 | 6.8 | 2.0 | 7.4 | 2.2 | 3.4 | 1.4 | 4.3 | 1.7 | 1.3 | 2.0 | 0.7 | 1.1 | 1.0 | 1.5 | 1.0 | 1.5 | 0.1 | 1.2 | 1.8 |  |
| Vitamin B6 (mg) | 0.06 | 0.12 | 0.06 | 0.12 | 0.06 | 0.12 | 0.15 | 0.05 | 0.13 | 0.04 | 0.05 | 0.02 | 0.02 | 0.01 | 0.10 | 0.15 | 0.01 | 0.02 | 0.01 | 0.02 | Tr | Tr | Tr | 0.01 | 0.07 |  |
| Vitamin B12 (μg) | 0.9 | 1.9 | 0.9 | 1.9 | 0.8 | 1.7 | 2.4 | 0.7 | 1.3 | 0.4 | 0.6 | 0.2 | 0.5 | 0.2 | 0.2 | 0.3 | 0.3 | 0.5 | 0.3 | 0.5 | 0.3 | 0.5 | 0.5 | 1.0 | 1.4 |  |
| Folate (μg) | 8 | 16 | 9 | 19 | 9 | 19 | 31 | 9 | 56 | 17 | 22 | 9 | 36 | 14 | 18 | 27 | 10 | 15 | 18 | 27 | 16 | 24 | 15 | 15 | 15 |  |
| Pantothenate (mg) | 0.58 | 1.19 | 0.68 | 1.40 | 0.50 | 1.0 | 0.50 | 0.15 | 0.51 | 0.15 | 0.30 | 0.12 | 0.32 | 0.13 | 0.50 | 0.75 | 0.40 | 0.60 | 0.56 | 0.84 | 0.33 | 0.50 | 0.38 | 0.47 | N |  |
| Biotin (μg) | 2.5 | 5.2 | 3.0 | 6.2 | 2.5 | 5.2 | 4.4 | 1.3 | 3.8 | 1.1 | 5.1 | 2.0 | 8.1 | 3.2 | 2.6 | 3.9 | 1.1 | 1.7 | 1.5 | 2.3 | 2.3 | 3.5 | 0.6 | Tr | N |  |
| Vitamin C (mg) | 2 | 4 | 2 | 4 | 1 | 2 | Tr | Tr | Tr | Tr | Tr | Tr | Tr | Tr | 1 | 2 | 1 | 2 | 1 | 2 | 1 | 2 | Tr | Tr | Tr |  |
| Retinol (μg) | 36 | 74 | 19 | 39 | 1 | 2 | 364 | 109 | 266 | 80 | 64 | 26 | 39 | 16 | 28 | 42 | 36 | 54 | 8 | 12 | 10 | 15 | 82 | 3 | 3 |  |
| Carotene (μg) | 14 | 29 | 9 | 19 | Tr | Tr | 141 | 42 | 169 | 51 | 13 | 5 | 158 | 63 | 21 | 32 | Tr | Tr | Tr | Tr | Tr | Tr | Tr | Tr | Tr |  |
| Vitamin D (μg) | Tr | Tr | Tr | Tr | Tr | Tr | 0.3 | 0.1 | 0.1 | 0.0 | 0.0 | 0.0 | N | N | 0.0 | 0.0 | 0.1 | 0.2 | 0.1 | 0.2 | Tr | Tr | N | Tr | Tr |  |
| Vitamin E (mg) | 0.06 | 0.12 | 0.04 | 0.08 | Tr | Tr | 0.52 | 0.16 | 0.66 | 0.20 | 0.14 | 0.06 | N | N | 0.05 | 0.08 | 0.18 | 0.27 | Tr | Tr | 0.28 | 0.42 | 0.15 | Tr | Tr |  |
| Sodium (mg) | 42 | 87 | 43 | 89 | 44 | 92 | 723 | 217 | 720 | 216 | 250 | 100 | 260 | 104 | 80 | 120 | 58 | 87 | 63 | 95 | 62 | 93 | 60 | 37 | 33 |  |
| Potassium (mg) | 157 | 323 | 156 | 321 | 162 | 337 | 75 | 23 | 110 | 33 | 161 | 64 | 141 | 56 | 280 | 420 | 170 | 255 | 228 | 342 | 204 | 306 | 143 | 155 | 110 |  |
| Calcium (mg) | 120 | 247 | 120 | 247 | 125 | 260 | 739 | 222 | 840 | 252 | 127 | 51 | 121 | 48 | 200 | 300 | 122 | 183 | 162 | 243 | 140 | 210 | 140 | 127 | 87 |  |
| Magnesium (mg) | 11 | 23 | 11 | 23 | 11 | 23 | 29 | 9 | 39 | 12 | 13 | 5 | 11 | 4.4 | 19 | 29 | 13 | 20 | 16 | 24 | 15 | 23 | 11 | 12 | 8 |  |
| Phosphorus (mg) | 96 | 198 | 94 | 194 | 96 | 200 | 505 | 152 | 620 | 186 | 171 | 68 | 154 | 62 | 170 | 255 | 96 | 144 | 143 | 215 | 120 | 180 | 123 | 120 | 110 |  |
| Iron (mg) | 0.02 | 0.04 | 0.02 | 0.04 | 0.03 | 0 | 0.30 | 0.09 | 0.20 | 0.06 | Tr | Tr | Tr | Tr | 0.10 | 0.15 | 0.12 | 0.18 | 0.08 | 0.12 | 0.11 | 0.17 | 0.06 | 0.06 | 0.10 |  |
| Copper (mg) | Tr | Tr | Tr | Tr | Tr | Tr | 0.03 | 0.01 | 0.05 | 0.02 | Tr | Tr | Tr | Tr | Tr | Tr | Tr | Tr | 0.03 | 0.05 | Tr | Tr | 0.03 | 0.03 | 0.01 |  |
| Zinc (mg) | 0.5 | 1.0 | 0.4 | 0.8 | 0.5 | 1.0 | 4.1 | 1.2 | 2.8 | 0.8 | 0.6 | 0.2 | 1.1 | 0.4 | 0.7 | 1.1 | 0.4 | 0.6 | 0.6 | 0.9 | 0.5 | 0.8 | 0.4 | 0.6 | 0.3 |  |
| Chloride (mg) | 89 | 183 | 87 | 179 | 87 | 181 | 1040 | 312 | 1190 | 357 | 400 | 160 | 440 | 176 | 170 | 255 | 179 | 269 | 235 | 353 | 130 | 195 | 230 | 137 | 89 |  |
| Manganese (mg) | Tr | Tr | Tr | Tr | Tr | Tr | Tr | Tr | Tr | Tr | Tr | Tr | Tr | Tr | Tr | Tr | Tr | Tr | Tr | Tr | Tr | Tr | Tr | Tr | Tr |  |
| Selenium (μg) | 1 | 2 | 1 | 2 | 1 | 2 | 6 | 2 | 11 | 3 | 4 | 2 | 5 | 2 | 2 | 3 | 2 | 3 | 2 | 3 | 2 | 3 | Tr | 3 | 2 |  |
| Iodine (μg) | 31 | 64 | 30 | 62 | 30 | 62 | 30 | 9 | N | N | 24 | 10 | 17 | 7 | 63 | 95 | 27 | 41 | 34 | 51 | 48 | 72 | 17 | 23 | N |  |

^1^ Data taken from Finglas *et al.*, 2015 ^(^[^17^](#_ENREF_17)^)^

**Supplementary Table S3.** Association between milk and dairy consumption, and body fatness, in children: findings from a review of cross sectional studies

| **Reference** | **Details** | **Exposure** | **Results-Conclusion** | **Adjustment** | **Effect** |
| --- | --- | --- | --- | --- | --- |
| Rockett *et al.* (2001)*^(^*[*^176^*](#_ENREF_176)*^)^* | n=16882  Age: 9-14 y (7550 boys)  USA | Total dairy | Dairy intake was inversely associated with overweight in both boys and girls (P<0.05) | Age, energy intake | ↓ |
| Forshee and Storey (2003)^(^[^177^](#_ENREF_177)^)^ | n=3311 (1687 boys)  Age: 6-19 y  USA | Milk | Slight but significant inverse association between milk and BMI in girls but not boys (P<0.05 F) | Age, race, and gender | ↓ for girls  ↔ for boys |
| Novotny *et al.* (2004)*^(^*[*^178^*](#_ENREF_178)*^)^* | n=323 girls  Age: 9-14 y  USA | Dairy  Dietary Ca | There was an inverse association between Ca intake and iliac skinfold thickness (1 mg of total and dairy Ca was associated with 0.0025 mm (P=0.01) and 0.0026 mm (P=0.02) lower iliac skinfold thickness | Ethnicity, energy, weight, iliac skinfold | ↓ for dairy Ca |
| Barba *et al.* (2005)*^(^*[*^179^*](#_ENREF_179)*^)^* | n=884 (451 boys)  Age: 7.5 y  Italy | Milk (full-fat and reduced-fat) | There were inverse associations between milk consumption and age- and sex-specific BMI z-scores (P=0.005) and overweight. Whole milk consumption remained statistically significant but not for reduced-fat milk. | Age, gender, physical activity, birth weight and parental overweight and education | ↓ |
| Dixon *et al.* (2005)*^(^*[*^47^*](#_ENREF_47)*^)^* | n=342 (171 boys)  Age: 4-10 y  USA | Total dairy  Dietary Ca | There were no significant associations between dairy and dietary calcium intake with BMI and skinfold measures in all age groups of children | Age, gender, energy intake, and percentage energy from fat, | ↔ |
| Fiorito *et al.* (2006)*^(^*[*^180^*](#_ENREF_180)*^)^* | n=172 (girls)  Age: 11 y  USA | Dairy  Ca | Higher consumption of dairy products (≥3 serv/d) was related to lower body mass index z scores and body fat than lower dairy consumption (<3 serv/d). There were no relationship found between dairy intake and weight status in a subset of plausible diet reporters | Unadjusted | ↓ total sample  ↔ in a subset of plausible diet reporters |
| O’Connor *et al.* (2006)*^(^*[*^181^*](#_ENREF_181)*^)^* | n=1160  Age: 2-5 y  USA | Milk | There were no statistically or clinically significant associations between the amount or type of milk consumed and BMI | Age, gender, ethnicity, income, energy intake, physical activity | ↔ |
| LaRowe *et al.* (2007)*^(^*[*^182^*](#_ENREF_182)*^)^* | n=541  Age: 2-5 y  n=793  Age: 6-11 y  USA | Milk (full-fat and reduced-fat) | There was no significant difference in BMI between fruit juices, milk, sweetened drink, water clusters at 2-5 y. At 6-11 y, high-fat milk pattern with significant lower adjusted BMI than water, sweetened drink, or soda pattern (P<0.05) | Age, gender, ethnicity, income, birth weight, diet quality and physical activity | Milk (FF or R) vs other drinks:  ↔ in 2-5 y + R milk in 6-11 y  ↓ in FF milk 6-11 y |
| Moore *et al.* (2008)*^(^*[*^55^*](#_ENREF_55)*^)^* | n=6095  Age: 5-11 y  n=4520  Age: 12-16 y  USA | Dairy | At 5-11 y, there were no associations between dairy intake and anthropometric indices of body fat. At 12-16 y, highest dairy consumption had lower estimated levels of fat in both genders | Age, gender, socio-economic status, race/ethnicity, height and television watching. | ↔ 5-11 y olds  ↓ 12-16 y olds |
| Murphy *et al.* (2008)*^(^*[*^183^*](#_ENREF_183)*^)^* | n=1521  Age: 2-5 y  n=2097  Age: 6-11 y  n=3939  Age: 12-18 y  USA | Milk (plain, flavoured) | There were no significant differences in BMI and BMI z-scores between plain or flavoured milk drinkers, and non-milk drinkers, at 2-11 y. At 12-18 y, there was significantly higher BMI and BMI Z-scores in non- milk drinkers compared with the milk drinkers (>60 ml/d) (P<0.05) | Energy intake, age, gender | ↔ 2-11 y olds  ↓ 12-18 y olds |
| Almon *et al.* (2010)*^(^*[*^184^*](#_ENREF_184)*^)^* | n=298 (162 boys)  Age: 9-10 y  n=386 (190 boys)  Age: 15-16 y  Sweden | Dairy (milk, soured milk, yoghurt and cheese) | Lactose intolerant participants ate significantly less dairy (P=0.001), no difference in %BF.  Lactase persistence linked to an overall higher milk and dairy intake, but not linked to higher body fat mass | Genotype, gender, age | ↔ |
| Bradlee *et al.* (2010)*^(^*[*^185^*](#_ENREF_185)*^)^* | n=3761 (1866 boys)  Age: 5-11 y  n=1803 (853 boys)  Age: 12-16 y  USA | Dairy | There were no significant associations at 5-11 y. At 12-16 y, dairy intake was negatively associated with WC (P=0.001 F/P=0.015 M) and SSFT (P=0.006 F/P=0.047 M). At 12-16 y milk (P=0.004) and cheese (P=0.020) intakes were negatively associated with WC≥85^th^ %ile in NHANES III, and milk only (P=0.008) in NHANES '99-'02 | Age, height, race/ethnicity, Tanner stage, television viewing and parental education | ↔ 5-11 y olds  ↓ 12-16 y olds |
| Eriksson and Strandvik (2010)^(^[^186^](#_ENREF_186)^)^ | n=114 (62 boys)  Age: 8 y  Sweden | Milk | There was significantly lower BMI in children drinking full-fat (not reduced fat) milk regularly vs. seldom/never (P<0.001) | Gender, carbohydrates, type of milk, parental ethnicity and education, family income, gender and BMI | ↓ for full fat milk  ↔ for other milks |
| Wiley *et al.* (2010)*^(^*[*^30^*](#_ENREF_30)*^)^* | n=1493  Age: 2-4 y  n=2526  Age: 5-10 y  USA | Dairy  Milk | A dietary pattern characterized by greater milk intake, and to a lesser extent, dairy product intake, was associated with increased BMI, especially among preschool age children. Dairy and milk were positively associated with BMI %ile at 2-4 y (P<0.001, P<0.005); milk, not dairy, associated with BMI at 5-10 y (P<0.02) | Age, birth weight, ethnicity | ↑ for milk and dairy in pre-school  ↑ for milk ↔ for dairy in 5-10 y olds. |
| Abreu *et al.* (2012)*^(^*[*^187^*](#_ENREF_187)*^)^* | n=903 (370 boys)  Age: 15-16 y  Portugal | Dairy | After adjustments, ≥2 serv/d of dairy products was a negative predictor of AO (OR (95% CI): 0.22 (0.07, 0.63)) only in boys | Age, energy intake, protein, total fat, sat fat, sugar, dietary fiber, total Ca intake, calcium-to-protein ratio, parental education, pubertal stage, physical activity, and smoking | ↓ for boys  ↔ for girls |
| Abreu *et al.* (2012)*^(^*[*^188^*](#_ENREF_188)*^)^* | n=1001 (418 boys)  Age: 15-18 y  Portugal | Dairy | There was an inverse association between milk intake and both BMI and %BF only in girls (P=0.01 and P=0.03). No association between total dairy, yoghurt, cheese and BMI or %BF | Age, birth weight, energy intake, protein, total fat, sugar, dietary fiber, total Ca intake, low-energy reporters, parental education, pubertal stage, and physical activity | ↓ for girls  ↔ for boys |
| Al-Hazzaa *et al.* (2012)*^(^*[*^189^*](#_ENREF_189)*^)^* | n=2906 (1400 boys)  Age: 14-19 y  Saudi Arabia | Dairy  Milk | There were no associations between the frequency of milk consumption and likelihood of being overweight/obese in adolescents | Age | ↔ |
| Danyliw *et al.* (2012)*^(^*[*^190^*](#_ENREF_190)*^)^* | n=2150  Age: 2-5 y  n=3613 (1831 boys)  Age: 6-11 y  n=4275 (2255 boys)  Age: 12-18 y  Canada | Milk (plain, high fat (>2% fat)) | There were no associations between milk or high-fat milk cluster pattern and body weight in all age groups, with no increased risk for obesity | Age, gender, ethnicity, energy intake, physical activity, socioeconomic status | ↔ |
| Perez-Rodriguez *et al.* (2012)*^(^*[*^191^*](#_ENREF_191)*^)^* | n=192  Age: 8-10 y  Mexico | Dairy | The proportion of children consuming ≥3 serv/d of dairy products was higher (P<0.01) among the NW children (18.3%) than among the obese children (6.4%). The risk of obesity was lower with drinking ≥3 serv/d 3 dairy portions (OR (95% CI): 0.34 (0.11, 1.03)) | Unadjusted | ↓ |
| Bel-Serrat *et al.* (2013)*^(^*[*^192^*](#_ENREF_192)*^)^* | n=511 (49.9% boys)  Age: 12.5-17.5 y  Europe | Dairy | Dairy consumption (milk, yoghurt and milk- and yoghurt-based beverages) was inversely associated with waist circumference and sum of skinfolds in adolescents | Pubertal maturity, socio-economic status, MVPA, sedentary behaviours, daily energy intake and study centre | ↓ |
| Beck *et al.* (2013)*^(^*[*^193^*](#_ENREF_193)*^)^* | n=319 (47% boys)  Age: 8-10 y  Mexico | Milk (plain, flavoured, reduced fat, high fat (>2% fat)) | There were no associations between 1% milk and not-fat milk with obesity, although flavoured milk consumption was related to lower odds of obesity (OR (95% CI): 0.88 (0.80, 0.96)) P=0.004 | Age, physical activity (percentage of time in moderate-to-vigorous activity), maternal BMI, maternal acculturation, maternal occupational status, gender, fast-food consumption, weekly screen time, maternal education, maternal country of origin and household income. | ↔ |
| Coppinger *et al.* (2013)*^(^*[*^194^*](#_ENREF_194)*^)^* | n=248 (216 boys)  Age: 9-13 y  United Kingdom | Milk | There was no association of milk consumption and choice of beverage with BMI or BMI Z-score | Underreporting | ↔ |
| Fayet *et al.* (2013)*^(^*[*^195^*](#_ENREF_195)*^)^* | n=4487  Age: 2-16 y  Australia | Milk (plain and flavoured) | There were no differences in BMI and waist circumference between flavoured milk or exclusively plain milk drinkers and non-consumers of milk (P<0.05) | Age, gender | ↔ |
| Gunther *et al.* (2013)*^(^*[*^196^*](#_ENREF_196)*^)^* | n=101  Age: 8-13 y  USA | Dairy  Dietary Calcium | Dairy and Ca consumption was inversely associated with BMI percentile (P=0.005) but there were no associations in overweight or obese subjects | Age and energy intake | ↓ |
| Junaibi *et al.* (2013)*^(^*[*^197^*](#_ENREF_197)*^)^* | n=1440 (736 boys)  Age: 6-19 y  UAE | Dairy | Dairy consumption was inversely associated with the BMI percentile (a reduction in BMI by 2.52 percentile points with each additional daily dairy consumption) | Unadjusted | ↓ |
| Moschonis *et al.* (2013)*^(^*[*^198^*](#_ENREF_198)*^)^* | n=2073  Age: 9-13 y  Greece | Dairy | A lifestyle pattern characterized by a higher dairy consumption with a more adequate breakfast was associated with lower odds of obesity and/or increased fat mass levels | Gender, Tanner stage, parental BMI, socio-economic status and birth weight. | ↓ |
| Papandreou *et al.* (2013)*^(^*[*^199^*](#_ENREF_199)*^)^* | n=607  Age: 7-15 y  Greece | Milk (full-fat and reduced-fat) | Sugar beverage consumption was associated with higher odds of obesity (OR (95% CI): 2.57 (1.06, 3.38)) P=0.029 but 100% fruit juices and milk were not associated with obesity | Age, gender and income, energy intake and physical activity | ↔ |
| Abreu *et al.* (2014)*^(^*[*^200^*](#_ENREF_200)*^)^* | n=494 (208 boys)  Age: 15-18 y  Portugal | Total dairy | Milk consumption was inversely associated with CMRS in adolescents (OR (95% CI): 0.53 (0.30, 0.93)) P=0.019. There were no associations between CMRS and total dairy, yoghurt, and cheese intake | Yoghurt and cheese intake, under reporters, pubertal stage, gender | Note: outcome = CMRS.  ↓ for milk  $\leftrightarrow$for other dairy |
| Nasreddine *et al.* (2014)*^(^*[*^201^*](#_ENREF_201)*^)^* | n=868  Age: 6-19 y  Lebanon | Dairy/Milk | Higher consumption of milk and dairy was inversely associated with the risk of developing obesity, overweight (OR (95% CI): 0.56 (0.32, 0.98)) and abdominal adiposity | Unadjusted | ↓ |
| Milla Tobarra *et al.* (2014)*^(^*[*^202^*](#_ENREF_202)*^)^* | n=373 (177 boys)  Age: 9-11 y  Spain | Milk | Thinner boys consumed more milk drinks (2.97 ml/kg) and thinner girls more whole milk (5.43 ml/kg) than their overweight counterparts | Age, gender, cardiorespiratory  fitness | ↓ |
| Keast *et al.* (2015)*^(^*[*^203^*](#_ENREF_203)*^)^* | n=3821  Age: 8-18 y  USA | Dairy  Yoghurt | Consumption of yoghurt and dairy was not associated with higher body weight, subscapular skinfold thickness and waist circumference in children | Demographic and lifestyle factors, including physical activity, total energy intake, physical activity level, TV/computer/video game usage, and smoking and alcohol use | ↔ |
| Lopez Legarrea *et al.* (2015)*^(^*[*^204^*](#_ENREF_204)*^)^* | n=21385  Age: 14 ± 1 y  Chile | Dairy | There was lower dairy product consumption in the obese group relative to normal weight and overweight children. Breakfast consumption was associated with normal body weight while only 50% of overweight and obese children consumed breakfast | Gender | ↔ |
| Ghotboddin Mohammadi *et al.* (2015)*^(^*[*^205^*](#_ENREF_205)*^)^* | n=785  Age: 10-19 y  Iran | Dairy | Higher consumption of total dairy, low fat dairy, high fat dairy, milk, yoghurt and cheese was not associated with BMI, but was inversely associated with the risk of MetS and its components compared with the lowest quartile of consumption respectively | Age, gender, energy intake, BMI | ↔ |
| Moreno *et al.* (2015)*^(^*[*^132^*](#_ENREF_132)*^)^* | n=511  Age: 12.5-17.5 y  Europe | Dairy  Yoghurt | Higher consumption of milk, yoghurt and of milk- and yoghurt-based beverages was associated with lower body fat, lower risk for CVD, and higher cardiorespiratory fitness | Socioeconomic status, pubertal maturity, MVPA, sedentary behavior, and daily energy intakes | ↓ |
| O’Sullivan *et al.* (2015)*^(^*[*^206^*](#_ENREF_206)*^)^* | n=1613 (826 boys)  Age: 14 y  Australia | Dairy | There was an inverse association between dairy consumption and overweight or obesity despite the increased nutrient density due to dairy consumption. The association of dairy and obesity was attenuated after adjustment for energy intake | Gender, family income, mother’s highest school year, puberty stage and physical activity/screen use, total daily energy intake, potential misreporting | ↓ but ↔ when adjusted for energy intake |
| Yamborisut *et al.* (2015)*^(^*[*^207^*](#_ENREF_207)*^)^* | n=1570 (789 boys)  Age: 6-12 y  Thailand | Ca | Calcium consumption among Thai children was inversely associated with body weight, BMI and sum 4-skinfold thickness  However, there was no association between milk consumption from FFQ on body weight, BMI or sum 4-skinfolds | Body fat, waist circumference | ↓ (Ca) /  ↔ (milk) |
| Castro Burbano *et al.* (2016)*^(^*[*^208^*](#_ENREF_208)*^)^* | n=244 (girls)  Age: adolescents  Ecuador | Ca | Dietary Ca and to a lesser extent dairy consumption was inversely associated with total and abdominal adiposity and to the prevalence of overweight | Unadjusted | ↓ |
| Fallah *et al.* (2016)*^(^*[*^209^*](#_ENREF_209)*^)^* | n=13486 (6769 boys)  Age: 6-18 y  Iran | Milk (full-fat and reduced-fat) | Consumption of non-pasteurised milk and full fat pasteurised milk was related to reduced risk of overweight and obesity compared to reduced fat pasteurised milk. There was a reduced overweight and obesity risk with full fat milk compared to reduced fat milk consumption | Age, gender, physical activity level, screen time, birth weight, milk type in infancy, family history of HTN, and frequency of other food groups consumed, dairy consumption frequency | ↓ |
| Moschonis *et al.* (2016)*^(^*[*^210^*](#_ENREF_210)*^)^* | n=600  Age: 9–13 y  Greece | Milk | Milk consumption was positively related to the number of stages performed in the endurance run test (*β* = 0.10, P=0.017) and negatively with BMI (*β* = -0.10; P=0.014) | Age, gender, dietary energy intake, protein, fat, fibre, carbohydrate, Ca, riboflavin, zinc levels, total plasma total homocysteine (tHcy), methylmalonic acid (MMA) and total steps per day | ↓ |
| Nezami *et al.* (2016)*^(^*[*^29^*](#_ENREF_29)*^)^* | n=536 (262 boys)  Age: 12-18 y  USA | Dairy | Dairy consumption was positively related to growth in all adolescents and to WHtR, FM, and FFM but only in boys. Milk and cheese consumption were positively related to central adiposity and body composition only in males. There were null associations between any type of dairy and body composition in females | Age, site, ethnicity, education of mother, and energy intake, soda intake, physical activity, and milk substitutes intake | ↑ boys / ↔ girls |
| Vanderhout *et al.* (2016)*^(^*[*^211^*](#_ENREF_211)*^)^* | n=2745 (1448)  Age: 1-6 y  Canada | Milk (full-fat and reduced-fat) | Full fat milk consumption was associated with 0.72 lower (95% CI: 0.68, 0.76) zBMI in children compared with reduced-fat milk consumption | BMI z score; 25(OH)D, 25-hydroxyvitamin D, age, gender, date of serum collection, skin pigmentation, daily vitamin D supplementation, milk volume consumption, maternal ethnicity, screen, outdoor play, maternal BMI, sugar-sweetened beverage consumption, median neighborhood family income | ↓ |
| Beck *et al.* (2017)*^(^*[*^212^*](#_ENREF_212)*^)^* | n=145 (74 girls)  Age: 3 y  USA | Milk (full-fat and reduced-fat) | Severely obese children were less likely to have consumed any milk (79% vs 95%, P=0.007) and a tendency toward consuming skim milk compared to those who were not severely obese (8% vs 2%, P=0.07). Severely obese children consumed fewer mean grams of milk fat (5.3 vs 8.9, P=0.009) although the volume of milk did not differ | Gender, maternal BMI, maternal education level (less than or greater than a high school education), maternal marital status, mother's preferred language, and mother's total years in the United States, total calories, fat, and milk consumption | ↓ |
| Chew *et al.* (2017)*^(^*[*^213^*](#_ENREF_213)*^)^* | n=832 (366 boys)  Age: 15-16 y  Malaysia | Dairy | Dairy and milk intake did not differ in adolescents with normal WC and those with abdominal adiposity (P=0.193) | Unadjusted | ↔ |

AO, abdominal obesity; BF, body fat; BMI, body mass index; Ca, calcium; CaCO_3_, calcium carbonate; CI, confidence interval; CMRS, cardio metabolic risk factors; HTN, hypertension; SDS, standard deviation score; WC, waist circumference. SSFT, sum of skinfold thickness; F, females; M, males; MVPA, moderate to vigorous physical activity

↓ negative association between exposure (dairy) and a measure of body fatness

↔ null association between exposure (dairy) and a measure of body fatness

↑ positive association between exposure (dairy) and a measure of body fatness

**Supplementary References**

175. Food Standards Agency & Public Health England (2016) National Diet and Nutrition Survey: rolling programme (years 5 and 6 combined). https://wwwgovuk/government/statistics/ndns-results-from-years-5-and-6-combined (accessed August 2017).

176. Rockett HR, Berkey CS, Field AE, *et al.* (2001) Cross-sectional measurement of nutrient intake among adolescents in 1996. *Preventive Medicine* **33**, 27-37.

177. Forshee RA, Storey ML (2003) Total beverage consumption and beverage choices among children and adolescents. *International Journal of Food Sciences and Nutrition* **54** 297-307.

178. Novotny R, Daida YG, Acharya S, *et al.* (2004) Dairy intake is associated with lower body fat and soda intake with greater weight in adolescent girls. *Journal of Nutrition* **134**, 1905-1909.

179. Barba G, Troiano E, Russo P, *et al.* (2005) Inverse association between body mass and frequency of milk consumption in children. *British Journal of Nutrition* **93**, 15-19.

180. Fiorito LM, Ventura AK, Mitchell DC, *et al.* (2006) Girls' dairy intake, energy intake, and weight status. *Journal of the American Dietetic Association* **106**, 1851-1855.

181. O'Connor T, Yang SJ, Nicklas TA (2006) Beverage intake among preschool children and its effect on weight status. *Pediatrics* **118**, e1010-1018.

182. LaRowe TL, Moeller SM, Adams AK (2007) Beverage patterns, diet quality, and body mass index of US preschool and school-aged children. *Journal of the American Dietetic Association* **107**, 1124-1133.

183. Murphy MM, Douglass JS, Johnson RK, *et al.* (2008) Drinking flavored or plain milk is positively associated with nutrient intake and is not associated with adverse effects on weight status in US children and adolescents. *Journal of the American Dietetic Association* **108**, 631-639.

184. Almon R, Patterson E, Nilsson TK, *et al.* (2010) Body fat and dairy product intake in lactase persistent and nonpersistent children and adolescents. *Food Nutrition Research* **16**.

185. Bradlee M, Singer M, Qureshi M, *et al.* (2010) Food group intake and central obesity among children and adolescents in the Third National Health and Nutrition Examination Survey (NHANES III). *Public Health Nutrition* **13** 797-805.

186. Eriksson S, Strandvik B (2010) Food choice is reﬂected in serum markers and anthropometric measures in healthy 8-yr-olds. *European eJournal of Clinical Nutrition and Metabolism* **5**, e117-e224.

187. Abreu S, Santos R, Moreira C, *et al.* (2012) Association between dairy product intake and abdominal obesity in Azorean adolescents. *Eur J Clin Nutr* **6**, 830-835.

188. Abreu S, Santos R, Moreira C, *et al.* (2012) Milk intake is inversely related to body mass index and body fat in girls. *European Journal of Pediatrics* **171**, 1467-1474.

189. Al-Hazzaa Hazzaa M, Abahussain NA, Al-Sobayel HI, *et al.* (2012) Lifestyle factors associated with overweight and obesity among Saudi adolescents. *BMC Public Health* **12**.

190. Danyliw AD, Vatanparast H, Nikpartow N, *et al.* (2012) Beverage patterns among Canadian children and relationship to overweight and obesity. *Applied Physiology, Nutrition, and Metabolism/* **37**, 900-906.

191. Perez-Rodriguez M, Melendez G, Nieto C, *et al.* (2012) Dietary and Physical Activity / Inactivity Factors Associated with Obesity in School Aged Children *Advances in Nutrition* **3** 622S-628S.

192. Bel-Serrat S, Mouratidou T, Jiménez-Pavón D, *et al.* (2014) Is dairy consumption associated with low cardiovascular disease risk in European adolescents? Results from the HELENA Study. *Pediatric Obesity* **9**, 401-410.

193. Beck A, Tschann J, Butte NF, *et al.* (2013) Association of beverage consumption with obesity in Mexican American children. *Public Health Nutrition* **17**, 338-344.

194. Coppinger T, Jeanes Y, Mitchell M, *et al.* (2013) Beverage consumption and BMI of British schoolchildren aged years. *Public Health Nutrition* **16**, 9-13.

195. Fayet F, Ridges LA, Wright JK, *et al.* (2013) Australian children who drink milk (plain or flavored) have higher milk and micronutrient intakes but similar body mass index to those who do not drink milk. *Nutrition Research* **33**, 95-102.

196. Gunther CW, Branscum P, Kennel J, *et al.* (2013) Examination of the Relationship of Dairy Product Consumption and Dietary Calcium with Body Mass Index Percentile in Children. *International Journal of Child Health and Nutrition* **2**, 1-8.

197. Al Junaibi A, Abdulle A, Sabri S, *et al.* (2012) The prevalence and potential determinants of obesity among school children and adolescents in Abu Dhabi, United Arab Emirates. *International Journal of Obesity* **37**, 68-74.

198. Moschonis G, Kalliora A, Costarelli V, *et al.* (2013) Identification of lifestyle patterns associated with obesity and fat mass in children: the Healthy Growth Study. *Public Health Nutrition* **17**, 614-624.

199. Papandreou D, Andreou E, Heraclides A, *et al.* (2013) Is beverage intake related to overweight and obesity in school children? *Hippokratia* **17**, 42-46.

200. Abreu S, Moreira P, Moreira C, *et al.* (2014) Intake of milk, but not total dairy, yogurt, or cheese, is negatively associated with the clustering of cardiometabolic risk factors in adolescents. *Nutrition Research* **34**, 48-57.

201. Nasreddine L, Naja F, Akl C, *et al.* (2014) Dietary, Lifestyle and Socio-Economic Correlates of Overweight, Obesity and Central Adiposity in Lebanese Children and Adolescents. *Nutrients* **6**, 1038-1062.

202. Milla Tobarra M, Martínez-Vizcaíno V, Lahoz García N, *et al.* (2014) The relationship between beverage intake and weight status in children: The Cuenca study. *Nutrición hospitalaria* **30**, 818-824.

203. Keast DR, Hill Gallant KM, Albertson AM, *et al.* (2015) Associations between yogurt dairy calcium and vitamin D intake and obesity among US children aged years NHANES. *Nutrients* **7(3)**, 1577-1593.

204. Lopez-Legarrea P, Olivares PR, Almonacid-Fierro A, *et al.* (2015) Association between dietary habits and the presence of overweight/obesity in a sample of 21,385 Chilean adolescents. *Nutrición hospitalaria* **31**, 2088-2094.

205. Ghotboddin Mohammadi S, Mirmiran P, Bahadoran Z, *et al.* (2015) The Association of Dairy Intake With Metabolic Syndrome and Its Components in Adolescents: Tehran Lipid and Glucose Study. *International Journal of Endocrinology and Metabolism* **13**, e25201.

206. O'Sullivan T, Bremner A, Bremer H, *et al.* (2014) Dairy product consumption, dietary nutrient and energy density and associations with obesity in Australian adolescents. *Journal of Human Nutrition and Dietetics* **28**, 452-464.

207. Yamborisut U, Wimonpeerapattana W, Rojroongwasinkul N, *et al.* (2015) Calcium intake in relation to body mass index and fatness in Thai school-aged children. *Open Journal of Pediatrics* **5**, 104-112.

208. Castro Burbano J, Fajardo Vanegas P, Robles Rodríguez J, *et al.* (2016) Relationship between dietary calcium intake and adiposity in female adolescents. *Endocrinology and Nutrition* **63**, 58-63.

209. Fallah Z, Kazemi E, Mollagh ME, *et al.* (2016) Risk of obesity and elevated blood pressure in relation to the type of milk consumed by children and adolescents: The CASPIAN-IV study. *Journal of Current Research in Science* **4**, 152-160.

210. Moschonis G, van den Heuvel E, Mavrogianni C, *et al.* (2016) Associations of milk consumption and vitamin B₂ and Β_12_ derived from milk with fitness, anthropometric and biochemical indices in children. The Healthy Growth Study. *Nutrients* **8**, E634.

211. Vanderhout S, Birken C, Parkin P, *et al.* (2016) Relation between milk-fat percentage, vitamin D, and BMI z score in early childhood. *Am J Clin Nutr* **104**, 1657-1664.

212. Beck A, Heyman M, Chao C, *et al.* (2017) Full fat milk consumption protects against severe childhood obesity in Latinos. *Preventive Medicine Reports* **8**, 1-5.

213. Chew WF, Leong PP, Yap SF, *et al.* (2017) Risk factors associated with abdominal obesity in suburban adolescents from a Malaysian district. *Singapore Medical Journal* doi: 10.11622/smedj.2017013 [Epub ahead of print].
